# Structural Variation of Plastomes Provides Key Insight Into the Deep Phylogeny of Ferns

**DOI:** 10.3389/fpls.2022.862772

**Published:** 2022-05-02

**Authors:** Xin-Yu Du, Li-Yaung Kuo, Zheng-Yu Zuo, De-Zhu Li, Jin-Mei Lu

**Affiliations:** ^1^Germplasm Bank of Wild Species, Kunming Institute of Botany, Chinese Academy of Sciences, Kunming, Yunnan, China; ^2^Institute of Molecular and Cellular Biology, National Tsing Hua University, Hsinchu, Taiwan

**Keywords:** leptosporangiates, Hymenophyllales, Gleicheniales, structural synapomorphies, large inversion, IR boundary, gene loss

## Abstract

Structural variation of plastid genomes (plastomes), particularly large inversions and gene losses, can provide key evidence for the deep phylogeny of plants. In this study, we investigated the structural variation of fern plastomes in a phylogenetic context. A total of 127 plastomes representing all 50 recognized families and 11 orders of ferns were sampled, making it the most comprehensive plastomic analysis of fern lineages to date. The samples included 42 novel plastomes of 15 families with a focus on Hymenophyllales and Gleicheniales. We reconstructed a well-supported phylogeny of all extant fern families, detected significant structural synapomorphies, including 9 large inversions, 7 invert repeat region (IR) boundary shifts, 10 protein-coding gene losses, 7 tRNA gene losses or anticodon changes, and 19 codon indels (insertions or deletions) across the deep phylogeny of ferns, particularly on the backbone nodes. The newly identified inversion V5, together with the newly inferred expansion of the IR boundary R5, can be identified as a synapomorphy of a clade composed of Dipteridaceae, Matoniaceae, Schizaeales, and the core leptosporangiates, while a unique inversion V4, together with an expansion of the IR boundary R4, was verified as a synapomorphy of Gleicheniaceae. This structural evidence is in support of our phylogenetic inference, thus providing key insight into the paraphyly of Gleicheniales. The inversions of V5 and V7 together filled the crucial gap regarding how the “reversed” gene orientation in the IR region characterized by most extant ferns (Schizaeales and the core leptosporangiates) evolved from the inferred ancestral type as retained in Equisetales and Osmundales. The tRNA genes *trnR-ACG* and *trnM-CAU* were assumed to be relicts of the early-divergent fern lineages but intact in most Polypodiales, particularly in eupolypods; and the loss of the tRNA genes *trnR-CCG, trnV-UAC*, and *trnR-UCU* in fern plastomes was much more prevalent than previously thought. We also identified several codon indels in protein-coding genes within the core leptosporangiates, which may be identified as synapomorphies of specific families or higher ranks. This study provides an empirical case of integrating structural and sequence information of plastomes to resolve deep phylogeny of plants.

## Introduction

Variations in the genomic structure of plastids, particularly large inversions or large boundary shifts of invert repeat regions (IR), are rare and complex and therefore less prone to homoplasy than nucleotide mutations (Palmer and Stein, 1986; Wolf et al., 2009). Actually, structural variation in the plastid genome has been used to infer the deep phylogeny of plants (Palmer and Stein, 1986) even earlier than DNA sequences, such as the plastid gene *rbcL* (Chase et al., 1993; Hasebe et al., 1995). Using physical and gene mapping methods, Raubeson and Jansen (1992) identified a 30-kb inversion shared by ferns (*Equisetum, Psilotum*, and *Osmunda*) and seed plants, while lycophytes (*Lycopodium*) shared the same gene orientation with the liverworts. Meanwhile, Stein et al. (1992) identified a nearly inverted gene order in the IR regions of tree ferns (Cyatheales) and polypod ferns (Polypodiales). Plastid genome sequencing of pteridophytes (ferns) did not begin until Wakasugi et al. (1998) released the first fern plastid genome, *Psilotum nudum* (L.) P. Beauv (Psilotaceae). They identified a 4.5-kb inversion (from *trnT-GGU* to *trnG-GCC*) in the large single-copy region (LSC) and detected the loss of the first intron in the *rps12* gene in *Psilotum*. Wolf et al. (2003) published the second fern plastome, *Adiantum capillus-veneris* L. (Pteridaceae), verified the 4.5-kb inversion in ferns and the inverted orientation of IRs in Polypodiales, and detected the loss of *psaM* and *trnK* genes in *Adiantum*.

With the accumulation of new plastome data (Roper et al., 2007; Gao et al., 2009, 2011, 2013; Wolf et al., 2009, 2010; Karol et al., 2010; Grewe et al., 2013; Kim et al., 2014), and the development of phylogenetic research (Schuettpelz and Pryer, 2007; Rai and Graham, 2010; Rothfels et al., 2015; Testo and Sundue, 2016; Qi et al., 2018; Shen et al., 2018), the evolutionary patterns of structural variations in fern plastomes became increasingly clear. Several studies summarized the patterns of fern plastome variation, including inversions, IR boundary shifts, and gene content (Wolf et al., 2011; Wolf and Karol, 2012; Kuo et al., 2018; Lehtonen and Cardenas, 2019). The study of Kuo et al. (2018) provided an order-level framework of plastid variations across ferns and with further analyses of Hymenophyllaceae, while some recent studies focused on plastome variations within specific lineages, such as Schizaeaceae (Labiak and Karol, 2017), Pteridaceae (Robison et al., 2018), and Polypodiaceae (Wei et al., 2021).

In the past two decades, pteridologists have endeavored to establish a convincing phylogenetic framework of ferns mainly using plastid sequences and nuclear genes (Pryer et al., 2004; Schuettpelz and Pryer, 2007; Rai and Graham, 2010; Lu et al., 2015; Rothfels et al., 2015; PPG I, 2016; Qi et al., 2018; Shen et al., 2018; Du et al., 2021). Nevertheless, the phylogenetic positions of some fern orders, such as Equisetales, Marattiales, Hymenophyllales, and Gleicheniales, are still controversial (Rothfels et al., 2015; Testo and Sundue, 2016; Lehtonen et al., 2017; Kuo et al., 2018; Qi et al., 2018; Shen et al., 2018; Lehtonen and Cardenas, 2019). In particular, the monophyly of Gleicheniales has been questioned by Qi et al. (2018) and Shen et al. (2018) by using nuclear genes. Previous studies revealed that the gene order and IR boundaries in some fern lineages such as Gleicheniales, Hymenophyllales, Marattiales, and Osmundales were quite dynamic (Grewe et al., 2013; Kuo et al., 2018; Lehtonen and Cardenas, 2019), particularly in Hymenophyllales (Kuo et al., 2018). However, due to insufficient plastome sequences and the lack of a reliable phylogenetic framework, the evolutionary pathway of inversions and IR boundary shifts among these lineages is still not clear. In particular, complete plastome sequences from Dipteridaceae and Matoniaceae are lacking. Wolf et al. (2010) proposed a two-step hypothesis to explain how the “reversed” gene orientation in IR regions of Schizaeales and the core leptosporangiates that were found nearly 30 years ago (Stein et al., 1992) evolved from the ancestral type in Hymenophyllales and Osmundales. However, the “intermediate” type they hypothesized had not been uncovered yet.

Facilitated by next-generation DNA sequencing technology (NGS), the number of published fern plastomes has increased rapidly in the past decade. There have been more than 600 fern plastome records in GenBank to date that cover most fern families (https://www.ncbi.nlm.nih.gov; last accessed on 31 December 2021). In this study, we aim to investigate the structural variation of fern plastome, including large inversion, IR boundary shift, tRNA gene loss or anticodon change, protein-coding gene or intron loss, and insertion or deletion (indels) of coding sequences (CDS) in an evolutionary context, based on comprehensive plastomic sampling that covers all fern families, and with analyses of structural variation identification and phylogeny reconstruction.

## Materials and Methods

### Sampling, DNA Extraction, Sequencing, and Plastome Assembly

Plastome sequences of 124 taxa, representing all 50 recognized families and 11 orders of ferns (following Liu et al., 2013; PPG I, 2016; Zhou et al., 2018), were gathered in this study, which includes 42 novel plastomes from 15 families with a focus on Hymenophyllales and Gleicheniales (Table 1; Supplementary Table 1), plus 82 plastomes accessed *via* GenBank (Table 1). Plastomes of two seed plants and one lycophyte were employed as outgroups (Table 1).

**Table 1:** The plastome data used in this study.

**No**.	**Family**	**Species**	**GenBank No**.	**Reference**
1	Lycopodiaceae	*Lycopodium clavatum* L.	NC040994	Mower et al., 2019
2	Amborellaceae	*Amborella trichopoda* Baill.	NC005086	Goremykin et al., 2003
3	Cycadaceae	*Cycas revoluta* Thunberg	NC020319	Li et al., unpublished
4	Equisetaceae	*Equisetum arvense* L.	NC014699	Karol et al., 2010
5	Equisetaceae	*Equisetum hyemale* L.	NC020146	Grewe et al., 2013
6	Ophioglossaceae	*Mankyua chejuensis* B.Y.Sun, M.H.Kim and C.H.Kim	NC017006	Kim and Kim, 2018
7	Ophioglossaceae	*Ophioglossum californicum* Prantl	NC020147	Grewe et al., 2013
8	Ophioglossaceae	*Sceptridium ternatum*	KM817789	Kim and Kim, 2018
9	Psilotaceae	*Psilotum nudum* (L.) P. Beauv.	NC003386	Wakasugi et al., 1998
10	Psilotaceae	*Tmesipteris elongata* Danguy	KJ569699	Zhong et al., 2014
11	Marattiaceae	*Angiopteris evecta* (G. Forst.) Hoffm.	NC008829	Roper et al., 2007
12	Marattiaceae	*Christensenia aesculifolia* (Blume) Maxon	NC044756	Liu et al., 2019
13	Marattiaceae	*Ptisana fraxinea* (Sm.) Murdock	OM419377	this study
14	Osmundaceae	*Osmunda japonica* Thunb.	OM419346	this study
15	Osmundaceae	*Osmundastrum cinnamomeum* (L.) C. Presl	OM419368	this study
16	Hymenophyllaceae	*Callistopteris apiifolia* (C. Presl) Copel.	OM419359	this study
17	Hymenophyllaceae	*Cephalomanes javanicum* (Blume) Bosch	OM419357	this study
18	Hymenophyllaceae	*Crepidomanes latealatum* (Bosch) Copel.	OM419367	this study
19	Hymenophyllaceae	*Hymenophyllum* aff. *dependens* C.V. Morton	OM419371	this study
20	Hymenophyllaceae	*Hymenophyllum badium* Hook. and Grev.	OM419373	this study
21	Hymenophyllaceae	*Hymenophyllum crassipetiolatum* Stolze	OM419354	this study
22	Hymenophyllaceae	*Hymenophyllum barbatum* (Bosch) Baker	OM419349	this study
23	Hymenophyllaceae	*Hymenophyllum holochilum* (Bosch) C. Chr.	NC039753	Kuo et al., 2018
24	Hymenophyllaceae	*Hymenophyllum pallidum* Ebihara and K.Iwats.	OM419369	this study
25	Hymenophyllaceae	*Hymenophyllum polyanthos* Bosch	OM419374	this study
26	Hymenophyllaceae	*Hymenophyllum sibthorpioides* (Willd.) Kuhn	OM419366	this study
27	Hymenophyllaceae	*Trichomanes siamense* Christ	OM419381	this study
28	Hymenophyllaceae	*Trichomanes trollii* Bergdolt	NC041122	Lehtonen, 2018
29	Hymenophyllaceae	*Vandenboschia auriculata* (Blume) Copel.	OM419344	this study
30	Hymenophyllaceae	*Vandenboschia speciosa* G.Kunkel	NC041000	Ruiz-Ruano et al., 2019
31	Gleicheniaceae	*Dicranopteris ampla* Ching and P.S. Chiu	OM419352	this study
32	Gleicheniaceae	*Dicranopteris pedata* (Houtt.) Nakaike	OM419363	this study
33	Gleicheniaceae	*Diplopterygium blotianum* (C. Chr.) Nakai	OM419345	this study
34	Gleicheniaceae	*Diplopterygium glaucum* (Thunb. ex Houtt.) Nakai	OM419358	this study
35	Gleicheniaceae	*Diplopterygium laevissimum* (H. Christ) Nakai	OM419370	this study
36	Gleicheniaceae	*Diplopterygium rufopilosum* (Ching and P.S. Chiu) Ching ex X.C. Zhang	OM419364	this study
37	Gleicheniaceae	*Sticherus truncatus* (Willd.) Nakai	OM419380	this study
38	Gleicheniaceae	*Stromatopteris moniliformis* Mett.	OM419372	this study
39	Dipteridaceae	*Cheiropleuria bicuspis* (Blume) C. Presl	OM419348	this study
40	Dipteridaceae	*Dipteris wallichii* (R. Br.) T. Moore	OM419347	this study
41	Matoniaceae	*Matonia pectinata* R. Br.	OM419375	this study
42	Lygodiaceae	*Lygodium japonicum* (Thunb.) Sw.	OM419353	this study
43	Lygodiaceae	*Lygodium microphyllum* (Cav.) R. Br.	OM419365	this study
44	Anemiaceae	*Anemia adiantifolia* (L.) Sw.	OM419342	this study
45	Schizaeaceae	*Actinostachys pennula* (Sw.) Hook.	KU764518	Labiak and Karol, 2017
46	Schizaeaceae	*Schizaea elegans* (Vahl) Sw.	NC035807	Labiak and Karol, 2017
47	Marsileaceae	*Marsilea crenata* C. Presl	KC536646	Gao et al., 2013
48	Marsileaceae	*Pilularia americana* A. Braun	OM419341	this study
49	Salviniaceae	*Azolla caroliniana* Willd.	MF177093	Robison et al., unpublished
50	Salviniaceae	*Salvinia cucullata* Roxb.	MF177095	Robison et al., unpublished
51	Cibotiaceae	*Cibotium barometz* (L.) J. Sm.	MT130589	Du et al., 2021
52	Culcitaceae	*Culcita coniifolia* (Hook.) Maxon	OM419350	this study
53	Cyatheaceae	*Alsophila costularis* Baker	NC044080	Wang et al., 2019
54	Cyatheaceae	*Sphaeropteris lepifera* (J. Sm. ex Hook.) R.M. Tryon	MN623357	Liu et al., 2020
55	Dicksoniaceae	*Dicksonia squarrosa* (G. Forst.) Sw.	KJ569698	Zhong et al., 2014
56	Loxsomataceae	*Loxsomopsis pearcei* (Baker) Maxon	OM419343	this study
57	Metaxyaceae	*Metaxya rostrata* (Kunth) C. Presl	OM419378	this study
58	Plagiogyriaceae	*Plagiogyria euphlebia* (Kunze) Mett.	NC046784	Yang et al., 2020
59	Plagiogyriaceae	*Plagiogyria subadnata* Ching	MN623362	Liu et al., 2020
60	Thyrsopteridaceae	*Thyrsopteris elegans* Kunze	OM419361	this study
61	Saccolomataceae	*Saccoloma elegans* Kaulf.	MT130580	Du et al., 2021
62	Cystodiaceae	*Cystodium sorbifolium* (Sm.) J. Sm.	MT130630	Du et al., 2021
63	Lonchitiaceae	*Lonchitis hirsuta* L.	MT130654	Du et al., 2021
64	Lonchitiaceae	*Lonchitis occidentalis* Baker	MT130627	Du et al., 2021
65	Lindsaeaceae	*Lindsaea cultrata* (Willd.) Sw.	MT130672	Du et al., 2021
66	Lindsaeaceae	*Odontosoria chusana* (L.) Masam.	MT130658	Du et al., 2021
67	Lindsaeaceae	*Osmolindsaea odorata* (Roxb.) Lehtonen and Christenh.	MT130576	Du et al., 2021
68	Lindsaeaceae	*Tapeinidium gracile* (Blume) v.A.v.R.	OM419362	this study
69	Dennstaedtiaceae	*Hypolepis punctata* (Thunb.) Mett.	MT130616	Du et al., 2021
70	Dennstaedtiaceae	*Microlepia obtusiloba* Hayata	MT130570	Du et al., 2021
71	Dennstaedtiaceae	*Monachosorum henryi* Christ	MT130593	Du et al., 2021
72	Pteridaceae	*Acrostichum aureum* L.	MT130571	Du et al., 2021
73	Pteridaceae	*Adiantum sinicum* Ching	MT130585	Du et al., 2021
74	Pteridaceae	*Calciphilopteris ludens* (Wall. ex Hook.) Yesilyurt and H. Schneid.	MT130590	Du et al., 2021
75	Pteridaceae	*Llavea cordifolia* Lag.	NC040216	Robison et al., 2018
76	Pteridaceae	*Pteris cretica* L.	MT130556	Du et al., 2021
77	Cystopteridaceae	*Acystopteris tenuisecta* (Blume) Tagawa	MT130692	Du et al., 2021
78	Cystopteridaceae	*Gymnocarpium oyamense* (Baker) Ching	MT130632	Du et al., 2021
79	Rhachidosoraceae	*Rhachidosorus consimilis* Ching	NC035862	Wei et al., 2017
80	Diplaziopsidaceae	*Diplaziopsis brunoniana* (Wall.) W. M. Chu	MT130567	Du et al., 2021
81	Diplaziopsidaceae	*Homalosorus pycnocarpos* (Spreng.) Pic. Serm.	NC035855	Wei et al., 2017
82	Desmophlebiaceae	*Desmophlebium lechleri* (Mett.) Mynssen, A. Vasco, Sylvestre, R.C. Moran and Rouhan	MT130626	Du et al., 2021
83	Hemidictyaceae	*Hemidictyum marginatum* (L.) C. Presl	MT130628	Du et al., 2021
84	Aspleniaceae	*Asplenium nidus* L.	MT130687	Du et al., 2021
85	Aspleniaceae	*Asplenium paucivenosum* (Ching) Bir	OM419382	this study
86	Aspleniaceae	*Asplenium wrightii* Eaton ex Hook.	OM419360	this study
87	Aspleniaceae	*Asplenium yoshinagae* Makino	OM419356	this study
88	Aspleniaceae	*Hymenasplenium obliquissiumum* (Hayata) Sugim.	MT130674	Du et al., 2021
89	Aspleniaceae	*Hymenasplenium obscurum* (Blume) Tagawa	OM419355	this study
90	Thelypteridaceae	*Christella appendiculata* (C. Presl) Holttum	NC035842	Wei et al., 2017
91	Thelypteridaceae	*Pseudophegopteris aurita* (Hook.) Ching	NC035861	Wei et al., 2017
92	Woodsiaceae	*Woodsia polystichoides* D. C. Eaton	MT130700	Du et al., 2021
93	Athyriaceae	*Athyrium foliolosum* T. Moore ex R. Sim	MT130638	Du et al., 2021
94	Athyriaceae	*Deparia viridifrons* (Makino) M. Kato	NC035846	Wei et al., 2017
95	Blechnaceae	*Blechnidium melanopus* (Hook.) T. Moore	MT130662	Du et al., 2021
96	Blechnaceae	*Woodwardia harlandii* Hook.	MT130602	Du et al., 2021
97	Onocleaceae	*Matteuccia struthiopteris* (L.) Tod.	MT130666	Du et al., 2021
98	Onocleaceae	*Pentarhizidium orientale* (Hook.) Hayata	MT130641	Du et al., 2021
99	Hypodematiaceae	*Hypodematium crenatum* (Forssk.) Kuhn	MT130540	Du et al., 2021
100	Didymochlaenaceae	*Didymochlaena truncatula* (Sw.) J. Sm.	MT130600	Du et al., 2021
101	Dryopteridaceae	*Bolbitis deltigera* (Bedd.) C. Chr.	MT130603	Du et al., 2021
102	Dryopteridaceae	*Ctenitis decurrentipinnata* (Ching) Ching	MT130665	Du et al., 2021
103	Dryopteridaceae	*Cyrtomium devexiscapulae* (Koidz.) Koidz. and Ching	NC028542	Lu et al., 2015
104	Dryopteridaceae	*Pleocnemia winitii* Holtt.	MT130681	Du et al., 2021
105	Lomariopsidaceae	*Cyclopeltis crenata* (Fee) C. Chr.	MT130541	Du et al., 2021
106	Lomariopsidaceae	*Lomariopsis longini* L. Y. Kuo and Y. H. Wu	MT130608	Du et al., 2021
107	Nephrolepidaceae	*Nephrolepis biserrata* (Sw.) Schott	MT130615	Du et al., 2021
108	Arthropteridaceae	*Arthropteris palisotii* (Desv.) Alston	MT130588	Du et al., 2021
109	Pteridryaceae	*Pteridrys cnemidaria* (Christ) C. Chr. and Ching	MT130579	Du et al., 2021
110	Tectariaceae	*Tectaria decurrens* (C. Presl) Copel.	MT130601	Du et al., 2021
111	Oleandraceae	*Oleandra wallichii* (Hook.) C. Presl	MT130650	Du et al., 2021
112	Davalliaceae	*Davallia assamica* (Bedd.) Baker	MT130637	Du et al., 2021
113	Polypodiaceae	*Drynaria quercifolia* (L.) J. Sm.	MT130596	Du et al., 2021
114	Polypodiaceae	*Lepisorus affinis* Ching	MT130664	Du et al., 2021
115	Polypodiaceae	*Loxogramme chinensis* Ching	MT130671	Du et al., 2021
116	Polypodiaceae	*Microgramma lycopodioides* (L.) Copel.	MT130699	Du et al., 2021
117	Polypodiaceae	*Micropolypodium sikkimensis* (Hieron.) X. C. Zhang	MT130599	Du et al., 2021
118	Polypodiaceae	*Pecluma dulcis* (Poir.) F.C. Assis and Salino	NC044685	Lehtonen and Cardenas, 2019
119	Polypodiaceae	*Platycerium wallichii* Hook.	MT130688	Du et al., 2021
120	Polypodiaceae	*Pyrrosia costata* (Wall. ex C. Presl) Tagawa and K. Iwats.	MT130646	Du et al., 2021
121	Polypodiaceae	*Selliguea chrysotricha* (C. Chr.) Fraser-Jenk.	OM419376	this study
122	Polypodiaceae	*Selliguea connexa* (Ching) S. G. Lu	MT130564	Du et al., 2021
123	Polypodiaceae	*Selliguea dareiformis* (Hook.) X. C. Zhang and L. J. He	MT130547	Du et al., 2021
124	Polypodiaceae	*Selliguea ebenipes* (Hook.) S. Linds.	OM419351	this study
125	Polypodiaceae	*Selliguea hastata* (Thunb.) H. Ohashi and K. Ohashi	OM419379	this study
126	Polypodiaceae	*Selliguea oxyloba* (Wall. ex Kunze) Fraser-Jenk.	MT130663	Du et al., 2021
127	Polypodiaceae	*Selliguea taeniata* Parris	MW876349	Wei et al., 2021

DNA samples were collected from silica-dried material or herbarium specimens of 42 individuals. DNA extraction, library preparation, and Illumina sequencing were facilitated by the Germplasm Bank of Wild Species, Kunming Institute of Botany, Chinese Academy of Sciences, following the routine of plastome sequencing from herbarium specimens (Zeng et al., 2018). Sequencing libraries were prepared using the NEBNext Ultra II DNA library Prep kit for Illumina (New England Biolabs). DNA was not fragmented by sonication, and the library was generated without any size selection. The final libraries were sequenced on Illumina HiSeq 2500 or X-Ten sequencing system (Illumina Inc.) to generate 1–4 Gb raw data of 150 bp paired-end reads. *De novo* assemblies were constructed with the GetOrganelle toolkit (Jin et al., 2020). Connection and reference-guided annotation were subsequently conducted using Bandage 0.8.1 (Wick et al., 2015) and Geneious 9.1.4 (Kearse et al., 2012). Our previously published plastomes were used as references (Du et al., 2021). To ensure all of the sampled plastomes are sufficiently complete to capture the structural changes, PCR approaches were used to further fill in some assembling gaps or verify the assembling results for the crucial samples such as *Matonia pectinata* R. Br., *Stromatopteris moniliformis* Mett., and *Cheiropleuria bicuspis* (Blume) C. Presl. Primer sequences used in PCR reactions are provided in Supplementary Table 2.

### Data Sets Construction and Phylogenetic Inference

The CDS of all 86 protein-coding genes were aligned by codon units using MAFFT (Katoh et al., 2005), and unreliably aligned regions were filtered using Gblocks v0.91b (Talavera and Castresana, 2007) with default parameters except half-gap positions were allowed. Then, the filtered data sets were concatenated into a supergene data set (the main data set) in Geneious 9.1.4 (Kearse et al., 2012), which holds an aligned length of 70,140 bp and an average GC content of 41% (varied from 33.9 to 45.1% among samples), and a sub data set excludes the third codon position that holds an aligned length of 46,760 bp and an average GC content of 44.4% (varied from 40.3 to 47.7% among samples). Maximum likelihood (ML) and Bayesian inference (BI) methods were used to infer phylogenetic relationships. ML analyses were conducted using IQ-tree 1.6.12 (Nguyen et al., 2015), with the GTR+F+R5 model selected by ModelFinder (Kalyaanamoorthy et al., 2017), the gene-partitioned model estimated by PartitionFinder2 (Lanfear et al., 2017), or the heterogeneous GHOST model (Crotty et al., 2020) GTR+H4, and 10,000 ultrafast bootstrap replicates. BI analyses were conducted using MrBayes 3.2.6 (Ronquist et al., 2012), with two runs of four Markov chain Monte Carlo (MCMC) chains for 10 million generations, and tree sampling frequency of 1,000 generations, and with gene-partitioned model estimated by PartitionFinder2. The first 25% of trees were discarded as burn-in, and the MCMC output was examined to check for convergence and to ensure that all the effective sample size (ESS) values were above 600.

### Gene Content and Structural Variation Investigation

For all resultant plastomes, the annotated protein-coding and tRNA genes were rechecked. Due to the prevalent RNA editing in fern plastomes (Lenz and Knoop, 2013; Ichinose and Sugita, 2017; Small et al., 2020), those genes with apparently abnormal start or stop codons, or internal stop codons were not judged as pseudogenes or lost. Alternatively, a relaxed criterion was used to judge the existence or absence of a gene, that is, a gene was treated as lost only if the integrity or similarity of open-reading frames (ORFs) of the target sequences dropped significantly (<70%) when compared with those normal gene sequences in phylogenetically related samples. For tRNA genes, the secondary structure and tRNA type and anticodon vs. isotype-specific model consistency were validated using tRNAscan-SE On-line (Lowe and Chan, 2016). Candidate tRNA sequences that passed secondary structure and tRNA type vs. isotype-specific model consistency validations were identified as true tRNA genes, and those sequences that possess altered anticodon were identified as anticodon changes in tRNA genes; otherwise, the candidate tRNA sequences were identified as hypothetical gene losses.

The structural variation and gene content of fern plastomes were investigated based on step-by-step alignment using Mauve (Darling et al., 2004) and MAFFT (Katoh et al., 2005). Plastomes of Equisetaceae, Ophioglossaceae, and Osmundaceae were inferred to retain the ancestral IR boundaries of land plants by Zhu et al. (2016). Our primary analyses revealed that plastomes of all early-diverging fern lineages (including Equisetaceae, Ophioglossaceae, Osmundaceae, as well as Psilotaceae, Marattiaceae, and Hymenophyllaceae), share identical gene order. The gene order of these extant lineages could represent the ancestral gene sequence of ferns, regardless of the variations in gene content and IR boundary. The number of plastid genes of Osmundaceae is the largest among ferns, and it is reasonable to infer that the plastomes of Osmundaceae retain the ancestral gene content of extant ferns based on the principle that specific plastid genes are easier to lose but difficult to obtain.

Plastomes of each fern family were aligned and compared first, then a representative plastome of each family was selected and aligned step-by-step, that is, taking the gene order, IR boundary, and gene content of plastomes in Osmundaceae as reference, plastomes used in an alignment were removed or added across the rooted phylogenetic tree of ferns, iteratively. Structural variation, including large inversion (V), IR boundary shift (R), and gene content variation, including the loss of the protein-coding gene or the intron (G), the loss of tRNA gene or anticodon change (T), was recorded, accordingly. Structural and gene content variations that occurred in a small number of samples were made a recheck against the assembling and annotation. Gene losses that occurred in only one sample were ignored in the subsequent analyses since these gene loss events are more likely to be autapomorphies of specific samples and have no phylogenetic significance in this study. Additionally, the CDS of each protein-coding gene was aligned rigorously using MAFFT (Katoh et al., 2005) to identify codon indels (insertion or deletion, D) in an evolutionary context. Finally, the parsimony principle is used to map familial- and higher-level changes onto the phylogenetic tree.

## Results

### Novel Plastomes

In this study, 42 complete or almost complete plastomes were generated, including the first plastome reports for 7 families, i.e., Anemiaceae, Culcitaceae, Dipteridaceae, Loxsomataceae, Matoniaceae, Metaxyaceae, and Thyrsopteridaceae (GenBank Nos OM419341-OM419382, Table 1). The detailed information, including average coverage, voucher information, and plastomic characteristics of newly generated plastomes, is provided in Supplementary Table 1. We identified several large fern plastomes due to extreme IR expansion toward the SSC region or large insertions in non-coding regions. The former includes *Asplenium yoshinagae* (Aspleniaceae, 186,828 bp, GenBank No. OM419356) and three samples of *Selliguea* (Polypodiaceae, 172,936 bp, 173,969 bp, and 178,147 bp, GenBank Nos. MT130547, OM419351, and MT130663), in which the SSC regions contain no gene or even with less than 50 bp in size (OM419351 and MT130663). The latter include *Desmophlebium lechleri* (Desmophlebiaceae, 169,014 bp, GenBank No. MT130626), *Matonia pectinata* (Matoniaceae, ~172,311 bp, GenBank No. OM419375), and *Saccoloma elegans* (Saccolomataceae, 174,044 bp, GenBank No. MT130580), which contain insertion fragments larger than 8 kb or even *ca*. 11.5 kb in *D. lechleri*.

### Familial-Level Phylogenetic Relationships of Ferns

**Figure 1:**
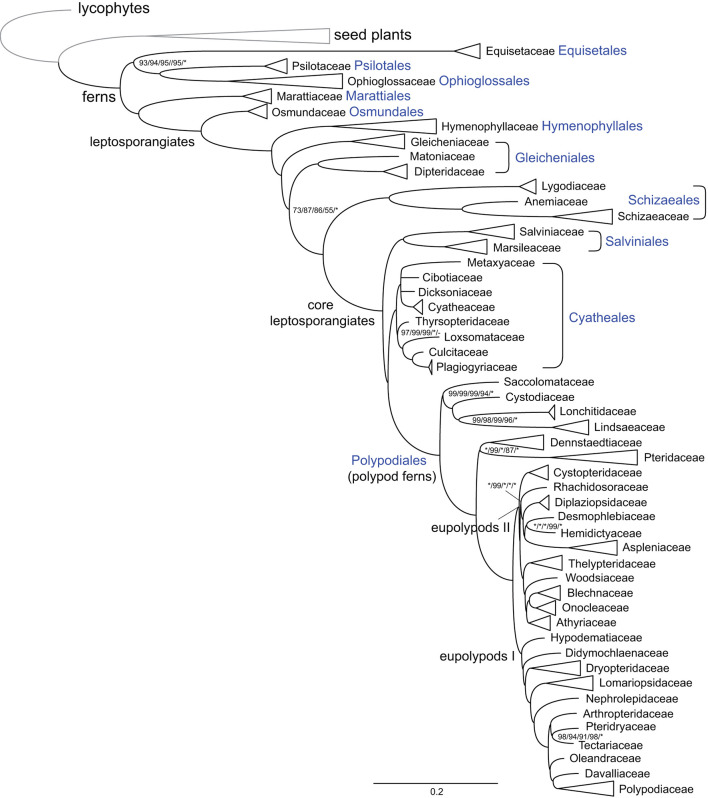
Schematic phylogram of ferns at the familial level. Tree topology and branch length indicated maximum likelihood (ML) analysis using CDS sequences and with the GTR+H4 model. The names of major deep nodes, orders, and families are indicated. Support values indicate ML analyses using CDS sequences and with the GTR+H4, gene-partitioned, or the GTR+F+R5 model, ML analysis using the first plus second codon position and with the GTR+F+R5 model, and Bayesian inference (BI) analysis using CDS sequences and with the gene-partitioned model, successively. Support values, including bootstrap support values (BS) and Bayesian confidence values (PP), are indicated along the branches, unless all BS and PP are 100% or 1.0. (*: 100% BS or 1.0 PP; –: support absent from the corresponding tree).

Phylogenetic analyses using different data sets, substitution models, or tree inference methods resolved mostly identical relationships among fern families and at higher levels, with strong support values on most nodes (Figure 1; Supplementary Figures 1–5). Equisetaceae were resolved as the sister clade of Ophioglossaceae plus Psilotaceae, and Marattiaceae were resolved as the sister clade of leptosporangiates. Dipteridaceae and Matoniaceae were resolved as sister to each other and together sister to a clade comprising Schizaeales and the core leptosporangiates with moderate to high support values (MLBS = 55–87) (Figure 1; Supplementary Figures 1–5). The relationships among four families of tree ferns, Cibotiaceae, Cyatheaceae, Dicksoniaceae, and Metaxyaceae, were not resolved (Figure 1; Supplementary Figures 1–5). The relationships among families in Polypodiales were in consensus with our previous study (Du et al., 2021).

### Structural Variation of Fern Plastomes

**Figure 2:**
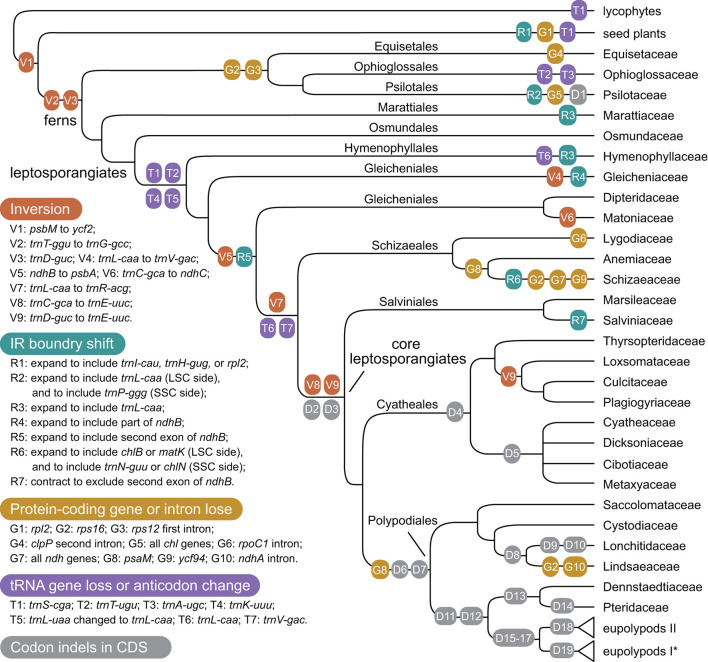
Evolution of plastome structure and gene content in ferns. Asterisks (*) indicates the loss of *rps16* gene (G2) was identified in Polypodiaceae; However, families in eupolypods I and II clades were collapsed for typographical convenience.

**Figure 3:**
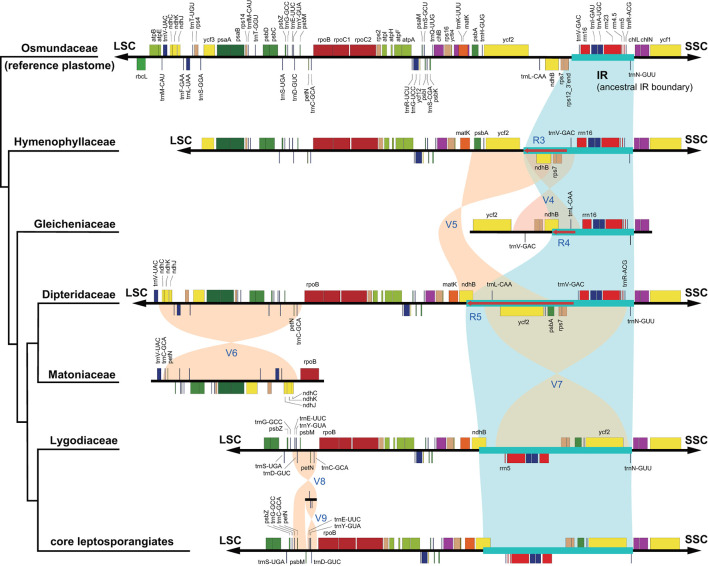
Major structural variations of plastomes across the phylogeny of leptosporangiate ferns.

**Figure 4:**
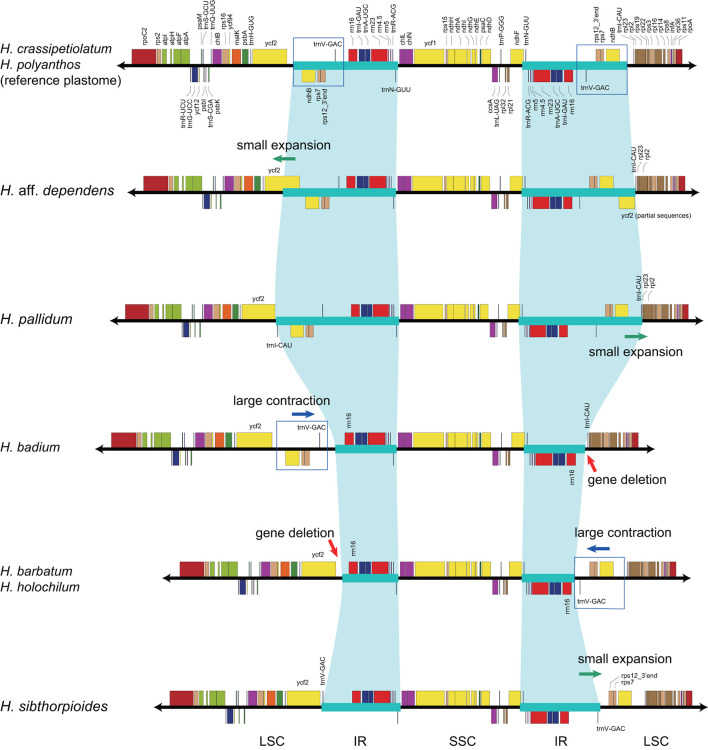
IR boundary shifts in *Hymenophyllum* plastomes.

With accurate gene annotation and step-by-step plastome comparison, a series of structural variations and gene content dynamics at the familial level and higher ranks were mapped onto the fern tree of life (Figure 2). We newly identified or validated 9 large inversions (V1–V9 in Figure 2), 7 large IR boundary shifts (R1–R7 in Figure 2), 10 losses of protein-coding genes (or gene families) or intron (G1–G10 in Figure 2), 6 losses of tRNA genes and one change of tRNA anticodon (T1–T7 in Figure 2), and 19 codon indels in protein-coding genes (D1–D19 in Figure 2) along the phylogenetic tree. A schematic diagram of plastome maps showing the major inversions (V4–V9) and IR boundary shifts (R3–R5) along the backbone of the fern phylogeny is provided in Figure 3, and a schematic diagram of plastome maps showing IR boundary shifts within *Hymenophyllum* (Hymenophyllaceae) is provided in Figure 4. In addition to those tRNA losses or anticodon change as illustrated in Figure 2 (T1–T7), we also identified five other tRNA gene losses with complex evolutionary patterns, and the corresponding validation results are provided in Supplementary Table 3. Position information and schematic screenshots for 19 identified codon indels are provided in Table 2; Supplementary Data 1, respectively. Moreover, we identified a few large inversions in Polypodiaceae, for example, *rrn5–rrn16* inversion in *Microgramma lycopodioides* (GenBank No. MT130699), and *ccsA–ndhF* inversion in five samples of *Selliguea* (GenBank Nos. MT130547, MT130663, OM419351, OM419376, and OM419379).

**Table 2:** The information of codon indels in protein-coding genes.

**No**.	**Node/Clade**	**Gene**	**Position in alignment**	**Length of insertion (+) or deletion (–)**
D1	Psilotaceae	*rps4*	118–255	+138
D2	core leptosporangiates	*matK*	193–207	−15
D3	core leptosporangiates	*ndhB*	1,354–1,362	+9
D4	Cyatheales	*ycf2*	3,610–3,633	−3
D5	Cibotiaceae, Metaxyaceae, Cyatheaceae, and Dicksoniaceae	*ycf2*	970–1,089	−108
D6	Polypodiales	*ycf2*	3,610–3,633	+24
D7	Polypodiales	*rps4*	469–477	−9
D8	Lindsaeaceae and Lonchitidaceae	*ndhB*	1,414–1,419	−6
D9	Lonchitidaceae	*rpoC2*	1,615–2,799	−1,032
D10	Lonchitidaceae	*ycf2*	2,527–2,610	−84
D11	Dennstaedtiaceae, Pteridaceae, and eupolypods	*rps4*	70–75	+6
D12	Dennstaedtiaceae, Pteridaceae, and eupolypods	*ycf2*	2,610–2,632	−6
D13	Dennstaedtiaceae and Pteridaceae	*ndhB*	205–213	−9
D14	Pteridaceae	*ycf2*	2,610–2,632	−9
D15	eupolypods	*ycf2*	862–873	−12
D16	eupolypods	*ycf2*	3,217–3,282	−63
D17	eupolypods	*ycf2*	5,128–5,133	+6
D18	eupolypods II	*chlL*	853–873	+21
D19	eupolypods I	*petA*	46–51	+6

*Schematic screenshots for the 19 codon indels are provided in Supplementary Data 1*.

## Discussion

### Structural Variation Provides Key Insights Into the Relationships Among Leptosporangiates

The phylogenetic relationships among Hymenophyllaceae, Gleicheniaceae, Dipteridaceae, and Matoniaceae have been controversial. Gleicheniaceae were resolved as the sister group to Dipteridaceae and Matoniaceae (Schuettpelz and Pryer, 2007; Rai and Graham, 2010; Lehtonen, 2011; Testo and Sundue, 2016), or sister to Hymenophyllaceae (Lehtonen et al., 2017; Qi et al., 2018; Shen et al., 2018; Lehtonen and Cardenas, 2019), or sister to a clade composed of Dipteridaceae, Schizaeales, and the core leptosporangiates (Rothfels et al., 2015; Liu, 2016). In this study, an effort was made to gather plastome sequences of all abovementioned related fern families, which enable us to investigate the phylogenetic relationships and structural evolution of the plastomes of these families. Our phylogenetic result unexceptionally supports the sister relationship between Dipteridaceae and Matoniaceae, and they together are sisters to a clade consisting of Schizaeales and the core leptosporangiates, which highlights the importance of adequate taxon sampling in phylogenetic analysis (Figure 1). Our results are congruent with previous phylogenetic studies using 25 low-copy nuclear genes (Rothfels et al., 2015) or 3 plastid genes (Liu, 2016), although they did not sample Matoniaceae and only received weak support on the relevant nodes.

The relationships among the leptosporangiate fern families provide a solid base to infer a number of newly identified or confirmed structural synapomorphies (Figures 2, 3). Taking the plastome of Osmundaceae as reference, we newly identified a unique inversion from *ndhB* to *psbA* in the LSC region (~16 kb, V5 in Figures 2, 3), together with a newly inferred IR expansion to include the second exon of the *ndhB* gene (R5 in Figures 2, 3) that was shared by a clade composed of Dipteridaceae, Matoniaceae, Schizaeales, and the core leptosporangiates. Meanwhile, taking the plastome of Osmundaceae as reference, an inversion from *trnV-GAC* to *trnL-CAA* (~12 kb, V4 in Figures 2, 3) (Wolf et al., 2010; Kim et al., 2014), together with an inferred IR expansion containing part of the *ndhB* gene (R4 in Figures 2, 3), was inferred to occur in the ancestors of Gleicheniaceae. Alternatively, if we assume that Gleicheniales are monophyletic (i.e., Gleicheniaceae are sister to Dipteridaceae plus Matoniaceae), an additional change event—reversed inversion V5—would be necessary to explain the observed gene order in Gleicheniaceae. Therefore, the inferred structural variation events (V4–V5 and R4–R5; Figures 2, 3), together with the phylogenetic results (Figure 1; Supplementary Figures 1–5), provide key insight into the paraphyly of Gleicheniales.

### Large Inversions in the Evolution of Fern Plastomes

The two inversions of V5 (together with the IR boundary shift R5) and V7 (~27 kb, *trnR-ACG* to *trnL-CAA* in the IR region) filled the key gap regarding how the “reversed” gene orientation in the IR region that characterized by Schizaeales and the core leptosporangiates evolved from the ancestral type as retained by Equisetales, Marattiales, Osmundales, and Hymenophyllales (Figures 2, 3), confirming the existence of the “intermediate” type in the plastomes of Dipteridaceae and Matoniaceae as suggested by Wolf et al. (2010). Interestingly, the “reversed” *rrn16-rrn23-rrn4.5-rrn5* gene cluster in the IR region is reversed again in *Microgramma lycopodioides* (Polypodiaceae), and some samples of Pteridaceae (Robison et al., 2018). Additionally, a newly identified inversion from *trnC-GCA* to *ndhC* in the LSC region (~24 kb, V6 in Figures 2, 3) was inferred to occur in the ancestors of Matoniaceae.

With much enhanced sampling of plastome data, we also confirmed a series of inversions found in previous studies and with more elaborate evolutionary patterns (Figures 2, 3). For instance, we confirmed the *ca*. 30 kb inversion (V1 in Figure 2) from *psbM* to *ycf2* that is shared by ferns and seed plants (Raubeson and Jansen, 1992); the *ca*. 4.5 kb inversion from *trnT-GGU* to *trnG-GCC* (V2 in Figure 2) (Wakasugi et al., 1998) and a small inversion covering *trnD-GUC* and its flanks (V3 in Figure 2) (Gao et al., 2009) that is shared by all ferns; and the inversion from *trnC-GCA* to *trnE-UUC* (~4 kb, V8 in Figures 2, 3) together with an inversion from *trnD-GUC* to *trnE-UUC* (~1 kb, V9 in Figures 2, 3) shared by the core leptosporangiates (Gao et al., 2009, 2013; Karol et al., 2010). Gao et al. (2011) detected the inversion of V9 in Plagiogyriaceae, while we further identified this inversion shared by a clade composed of Culcitaceae, Loxsomataceae, and Plagiogyriaceae in this study (Figure 2).

Liu et al. (2020) and Wei et al. (2021) reported an inversion from *ccsA* to *ndhF* in some samples of *Selliguea* (Polypodiaceae), we newly detected this inversion in more samples of *Selliguea*. According to our phylogenetic inference of *Selliguea*, the inversion shared by a sub-clade of *Selliguea* that excludes *S. connexa* and *S. taeniata* (Supplementary Figures 1–5).

### IR Boundary Shifts in the Evolution of Fern Plastomes

IR boundaries of the plastomes in eusporangiate ferns and some leptosporangiate ferns were quite dynamic, but due to the lack of stable phylogenetic framework and the insufficiency of plastome data, most of the evolutionary patterns of the IR boundary shifts inferred by previous studies were unclear and ambiguous (Karol et al., 2010; Grewe et al., 2013; Kuo et al., 2018). This study implied that the IR boundary shifts (expansions) had occurred independently in Hymenophyllaceae, Marattiaceae, and Psilotaceae (R2 and R3 in Figure 2), while Equisetaceae, Ophioglossaceae, and Osmundaceae seemingly retain the ancestral IR boundaries of land plants that host only the core rRNA/tRNA cluster (Zhu et al., 2016). The IR boundaries of Psilotaceae have been found to expand in both directions (R2 in Figure 2) (Wakasugi et al., 1998). The IR boundaries of Marattiaceae and Hymenophyllaceae were inferred to expand in the LSC direction to include three protein-coding genes (*rps12, rps7*, and *ndhB*) and *trnL-CAA* (R3 in Figure 2). Additionally, taking the plastome of *Hymenophyllum crassipetiolatum* and *H. polyanthos* as reference (GenBank Nos. OM419354 and OM419374), the IR boundaries of some *Hymenophyllum* samples (GenBank Nos. OM419373, OM419349, OM419366, and NC039753; Figure 4) seemed to contract substantially to exclude the three aforementioned protein-coding genes or even *trnV-GAC*, while small expansions occurred in some other samples (GenBank Nos. OM419369 and OM419371; Figure 4). By integrating the phylogenetic relationships (see Supplementary Figures 1–5) with gene orders of these samples (Figure 4), we infer that the IR boundary shifts in *Hymenophyllum* should have occurred multiple times, although more samples are needed to clarify an elaborate evolutionary pattern. Generally, the IR boundary shift patterns in Hymenophyllaceae we inferred were congruent with the results of Kuo et al. (2018). We also identified that the *trnL-CAA* gene was absent in all Hymenophyllaceae samples (T6 in Figure 2; Supplementary Table 2), and this tRNA gene was suggested to be pseudogenized in Hymenophyllaceae (Kuo et al., 2018).

The IR boundary shift patterns of Gleicheniaceae, and Dipteridaceae plus Matoniaceae were complex because each of the boundary shifts seemed to occur simultaneously with an inversion (Figures 2, 3). In Gleicheniaceae, the inversion V4 from *trnL-CAA* (LSC region) to *trnV-GAC* (ancestral IR region) was inferred to occur simultaneously with the IR expansion R4 that includes *trnL-CAA* and part of *ndhB* (Figures 2, 3). In Dipteridaceae and Matoniaceae, the inversion V5 from *psbA* (LSC region) to *ndhB* (ancestral LSC region) was inferred to occur simultaneously with the IR expansion R5 that includes the second exon of *ndhB* (Figures 2, 3). Notably, the IR boundaries of Dipteridaceae and Matoniaceae also retained by most extant leptosporangiates (Figures 2, 3), only with shifts in Schizaeaceae (R6 in Figure 2), and Salviniaceae (R7 in Figure 2), and in most samples of Polypodiaceae (except GenBank Nos. MT130599, MT130699, and NC044685); also in some samples of Aspleniaceae (GenBank Nos. OM419356 and OM419382), Dennstaedtiaceae (GenBank No. MT130570), Dryopteridaceae (GenBank No. MT130665), and Pteridaceae (GenBank Nos. MT130583 and MT130585).

Furthermore, our analyses of plastomes from all fern families did not reveal any untypical structures such as loss of IR found in green algae (Turmel et al., 2017) and land plants (Wicke et al., 2011), or DR (direct repeat) found in Selaginellaceae (lycophytes) (Zhang et al., 2019). Nevertheless, we found some cases with extremely small SSC regions due to IR expansion (e.g., some samples in Aspleniaceae and Polypodiaceae) or gene losses (e.g., Schizaeaceae).

### Contradictory Absence Patterns Among tRNA and Protein-Coding Genes

The losses of tRNA genes are common in fern plastomes, and some loss events showed clear evolutionary patterns while others appeared lost independently (Kuo et al., 2018; Lehtonen and Cardenas, 2019). Unfortunately, some researchers annotated tRNA genes only based on sequence similarity with reference plastomes, but they ignored the verification of anticodon, secondary structure, and isotype-specific model consistency, causing potential specious annotations. We performed anticodon versus isotype-specific model consistency validation for all the plastid tRNA genes, which enabled us to identify tRNA gene loss patterns in fern plastomes more accurately.

Our study showed that some tRNA genes were intact in early-divergent fern lineages and lost in other lineages, which can be identified as synapomorphies on specific deep phylogenetic nodes (Figure 2). Those tRNA genes that fall into this pattern that we detected were generally consistent with the result of Kuo et al. (2018). Specifically, we validated the losses of three tRNA genes (*trnS-CGA, trnT-UGU*, and *trnK-UUU*; T1, T2, and T4 in Figure 2) that correspond to *a, j*, and *m* in Kuo et al. (2018) and one anticodon change in tRNA (*trnL-UAA* to *trnL-CAA*; T5 in Figure 2; Supplementary Table 2) that corresponds to *k1* in Kuo et al. (2018) that is shared by all leptosporangiate ferns except Hymenophyllaceae; and loss of *trnV-GAC* gene (T7 in Figure 2) that corresponds to *x* in Kuo et al. (2018) that is shared by a clade consisting of Schizaeales and the core leptosporangiate ferns. Differed from the result of Kuo et al. (2018), the loss of *trnL-CAA* gene was inferred to occur in Hymenophyllaceae and Schizaeales plus the core leptosporangiates (T6 in Figure 2), respectively. While this gene was verified to be intact in all samples of Gleicheniaceae, Matoniaceae, and Dipteridaceae with the only exception of *Cheiropleuria bicuspis* (GenBank No. OM419348) in this study. In addition, we newly identified the loss of *trnA-UGC* gene in Ophioglossaceae (T3 in Figure 2) and *Actinostachys* (Schizaeaceae).

Some tRNA genes are intact in most polypod ferns (Polypodiales) but only occasionally found in samples outside of Polypodiales. In particular, *trnR-ACG* genes are intact in all samples of Polypodiales except Saccolomataceae and Lonchitidaceae but retained in some samples of Hymenophyllaceae, Gleicheniaceae, Matoniaceae, and Salviniales (Supplementary Table 2). Similarly, *trnM-CAU* genes are intact in most samples of Polypodiales (except some samples in Pteridaceae) and Cyatheales (except Cyatheaceae), but absent in all samples of Equisetaceae, Psilotaceae, Marattiaceae, Osmundaceae, Hymenophyllaceae, Gleicheniaceae, and Salviniaceae, and some samples of Ophioglossaceae and Schizaeaceae (Supplementary Table 3).

Some tRNA genes were usually annotated based on sequence similarity, but most of them could not pass the secondary structure, or tRNA type versus isotype-specific model consistency validation using tRNAscan-SE. In particular, the *trnR-CCG* genes were only validated in Hymenophyllaceae and some samples of Osmundaceae, Gleicheniaceae, and Dipteridaceae (Supplementary Table 3), which differs from the result of Kuo et al. (2018) based on ARAGORN (Laslett and Canback, 2004). The *trnR-UCU* genes were only validated in Hymenophyllaceae and Osmundaceae, and the *trnV-UAC* genes were only validated in a few samples of Pteridaceae (Supplementary Table 3). Our result implies that the loss of the tRNA genes in fern plastomes is more common and its pattern is more complex than previously estimated. Nevertheless, the failure in validation using tRNAscan-SE does not mean absolute loss or pseudogenization of these tRNA genes. Some of them may still be functional given the RNA editing process.

For the losses of protein-coding genes or introns, our results were largely congruent with previous studies (Grewe et al., 2013; Kim et al., 2014; Labiak and Karol, 2017; Kuo et al., 2018; Song et al., 2018). Different from the lost patterns of tRNA genes, the losses of protein-coding genes or introns in fern plastomes mainly occurred at or under the familial level, and thus can be identified as synapomorphies of specific lineages (Figure 2). The unique protein-coding gene loss event on the backbone of fern phylogeny is the loss of the *psaM* gene (G8 in Figure 2), which was initially identified in *Adiantum capillus-veneris* by Wolf et al. (2003), and we confirmed the loss of the *psaM* gene in Polypodiales and Schizaeales with broader sampling. We also confirmed that the loss of *rps16* gene and the first intron of *rps12* gene was shared by Equisetales, Ophioglossales, and Psilotales (G2 and G3 in Figure 2), which supports our phylogenetic inference (Figure 1). These gene loss events may not serve as strong evidence of the position of Equisetales, considering that gene loss is generally more likely to be homoplasy than large inversions, and that more other genes were also lost in Equisetales and Psilotales, respectively (G4 and G5 in Figure 2). We also identified the loss of *rps16* gene and *ndhA* intron in Lindsaeaceae (G2 and G10 in Figure 2), and the loss of the *rps16* gene in Polypodiaceae and some samples of Pteridaceae (GenBank Nos. MT130585 and MT130590). Furthermore, the novel gene *ycf94* discovered by Song et al. (2018) was confirmed to retain in nearly all sampled fern plastomes except those of Schizaeaceae (G9 in Figure 2).

We newly detected the losses of most (8 out of 11) *ndh* genes in *Stromatopteris moniliformis* (Gleicheniaceae, GenBank No. OM419372). The losses of *ndh* genes were also reported in the plastomes of Schizaeaceae (Labiak and Karol, 2017) (G7 in Figure 2), and many other distantly related seed plant lineages (Graham et al., 2017). Our study provides new evidence on the linkage between frequent protein-gene losses in certain lineages and their life history features, i.e., Both *Stromatopteris* and Schizaeaceae have evolved achlorophyllous, mycoheterotrophic forms during their gametophyte stage (Bierhorst, 1971; and for Schizaeaceae, reviewed in Ke et al., in this special issue). Generally, the plastome structure and gene content in core leptosporangiates are highly conserved, and only a few inversions, IR boundary shifts, and gene losses were detected. Besides the variations aforementioned, we also identified 19 codon indels in several nodes of the core leptosporangiates, especially in Polypodiales. These codon indels attributed mainly to the *ycf2, ndhB*, and *rps4* genes, and can also be identified as synapomorphies of specific lineages (Table 2; Supplementary Data S1).

### Other Issues

Structural changes in plastomes such as large inversions and IR boundary shifts also resulted in gene translocation into or out of IR, such as the genes *psbA, ycf2, rps7*, and 3′-end exon of the *rps12* gene. Some previous studies showed that those genes translocated into the IR region have decelerated substitution rates and elevated GC content (Li et al., 2016; Zhu et al., 2016). While studies focusing on Geraniaceae showed that the regional effect was not sufficient to explain the observed substitution rate and GC content variations (Guisinger et al., 2008; Weng et al., 2017). It seems that the rate heterogeneity among plastid genes is more likely the end product of locus- or lineage-specific and IR-dependent effects during the evolutionary history (Sloan et al., 2014; Weng et al., 2017; Liu et al., 2020). This study clarifies the evolutionary pathway of large inversions and IR boundary shifts, and gene or intron losses across all fern families, thus laying a solid foundation for further investigation of these issues.

Robison et al. (2018) analyzed sequence insertions in fern plastomes and identified three mobile elements (MORFFO 1/2/3), particularly in Pteridaceae. Subsequent studies expanded the searching for mobile ORFs across ferns and found that these mobile elements were also prevalent in the suborder Polypodiineae (Lehtonen and Cardenas, 2019), or Hymenophyllaceae (Kim and Kim, 2020), while less common in most other fern lineages. We examined the DNA sequences of the three MORFFOs using data mainly from Robison et al. (2018) and found that the similarity of these sequences are significantly lower than most plastid protein-coding sequences, e.g., ~55/59/63% pairwise identity for MORFFO 1/2/3 within Pteridaceae. Given the current insufficiency of nuclear or mitochondria genomes of ferns, large-scale identification and further analyses of these mobile elements across all fern lineages could be a tricky task. It would be more feasible to study mobile ORFs by focusing on specific lineages, e.g., Hymenophyllaceae, Pteridaceae, and Polypodiineae, with dense plastomic sampling.

## Conclusion

We newly identified or validated a series of evident plastomic structural synapomorphies for deep nodes on the fern tree of life, with the most comprehensive plastome sampling that covers all recognized fern families. This study provides valuable new plastomes to facilitate research on mobile ORF, phylogenetic and molecular dating analyses in ferns; and more importantly, provides an empirical case for integrating structural and sequence information of plastomes to resolve deep phylogeny of ferns. Furthermore, this study provides comprehensive backbone information for future plastomic research on ferns. For instance, the study of substitution rate analyses on the genes translocated into or out of IR regions (e.g., *ndhB, psbA, rps7, rps12*, and *ycf2*), or the genes with or without intron (e.g., *clpP, ndhA, rpoC1*, and *rps12*); or further structural investigation in some highly dynamic genera, such as *Asplenium* (Aspleniaceae), *Hymenophyllum* (Hymenophyllaceae), and *Selliguea* (Polypodiaceae).

## Data Availability Statement

The datasets presented in this study can be found in online repositories. The names of the repository/repositories and accession number(s) can be found in the article/Supplementary Material.

## Author Contributions

J-ML and D-ZL designed research. X-YD and Z-YZ performed research and analyzed data. L-YK sequenced the plastome of *Matonia*. X-YD, J-ML, L-YK, Z-YZ, and D-ZL wrote the paper. All authors contributed to the article and approved the submitted version.

## Funding

The study was supported by the National Natural Science Foundation of China (Grant Nos. 31970232 and 32000172), the Large-scale Scientific Facilities of the Chinese Academy of Sciences (2017-LSF-GBOWS-02), and the Technological Leading Talent Project of Yunnan (2017HA014).

## Conflict of Interest

The authors declare that the research was conducted in the absence of any commercial or financial relationships that could be construed as a potential conflict of interest.

## Publisher's Note

All claims expressed in this article are solely those of the authors and do not necessarily represent those of their affiliated organizations, or those of the publisher, the editors and the reviewers. Any product that may be evaluated in this article, or claim that may be made by its manufacturer, is not guaranteed or endorsed by the publisher.
