# Supplementary material for: Structural Variation of Plastomes Provides Key Insight Into the Deep Phylogeny of Ferns
Source: Front Plant Sci. 2022 May 2;13:862772. doi: 10.3389/fpls.2022.862772 (PMC9134734; doi:10.3389/fpls.2022.862772)
Supplement: Supplementary file 1 [file Data_Sheet_1.PDF]

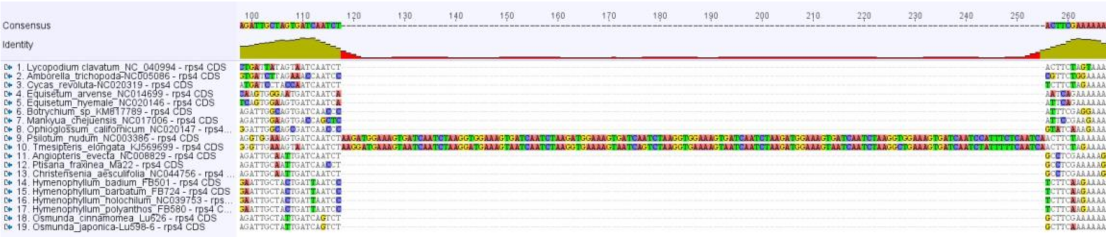

D1: Psilotaceae_rps4_118–255_+138 bp

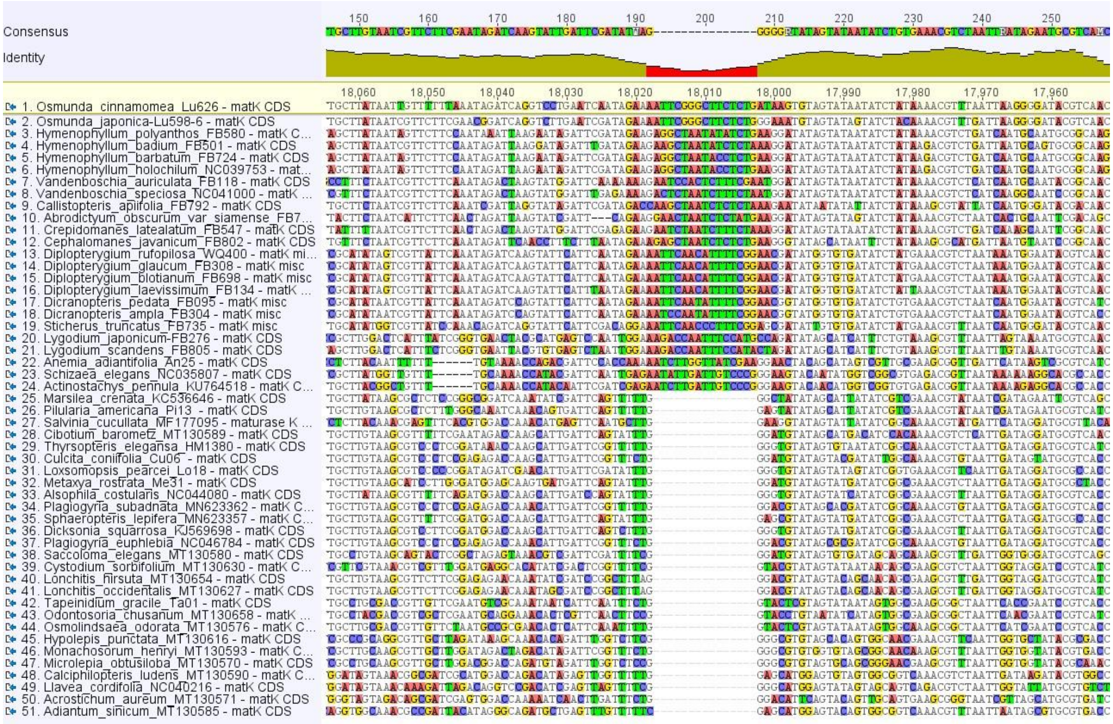

D2: core leptosporangiates_matK_193–207_-15 bp

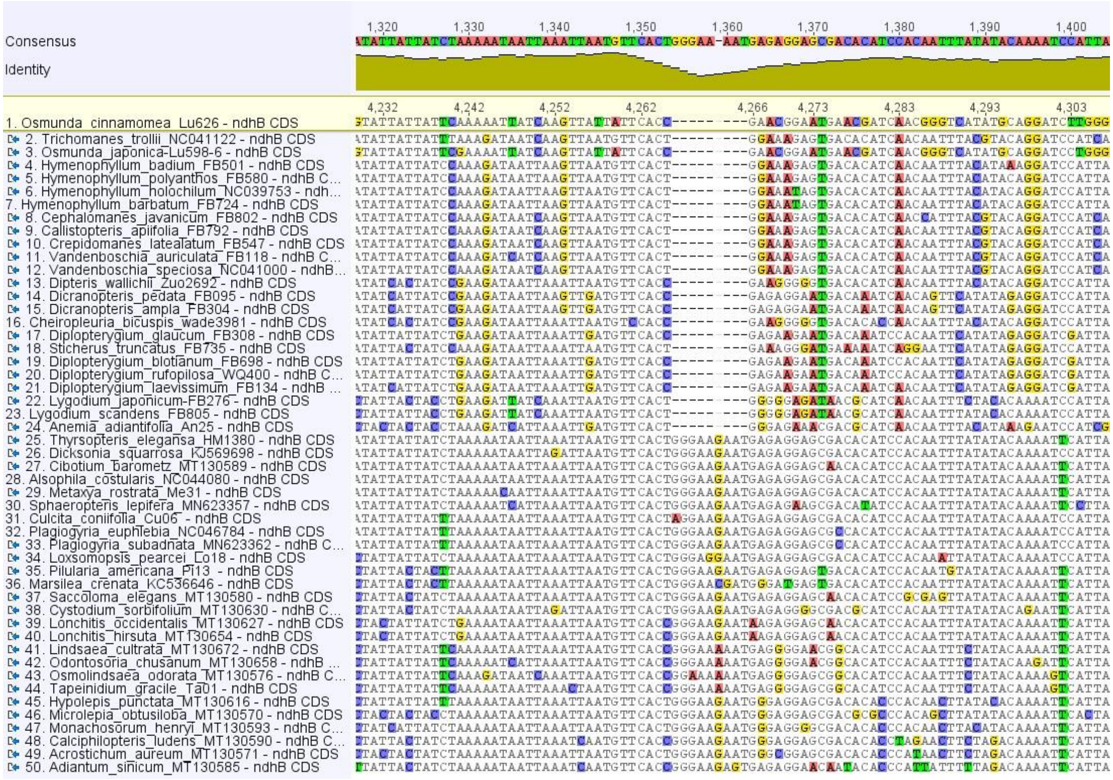

D3: core leptosporangiates_ndhB_1354–1362_+9 bp

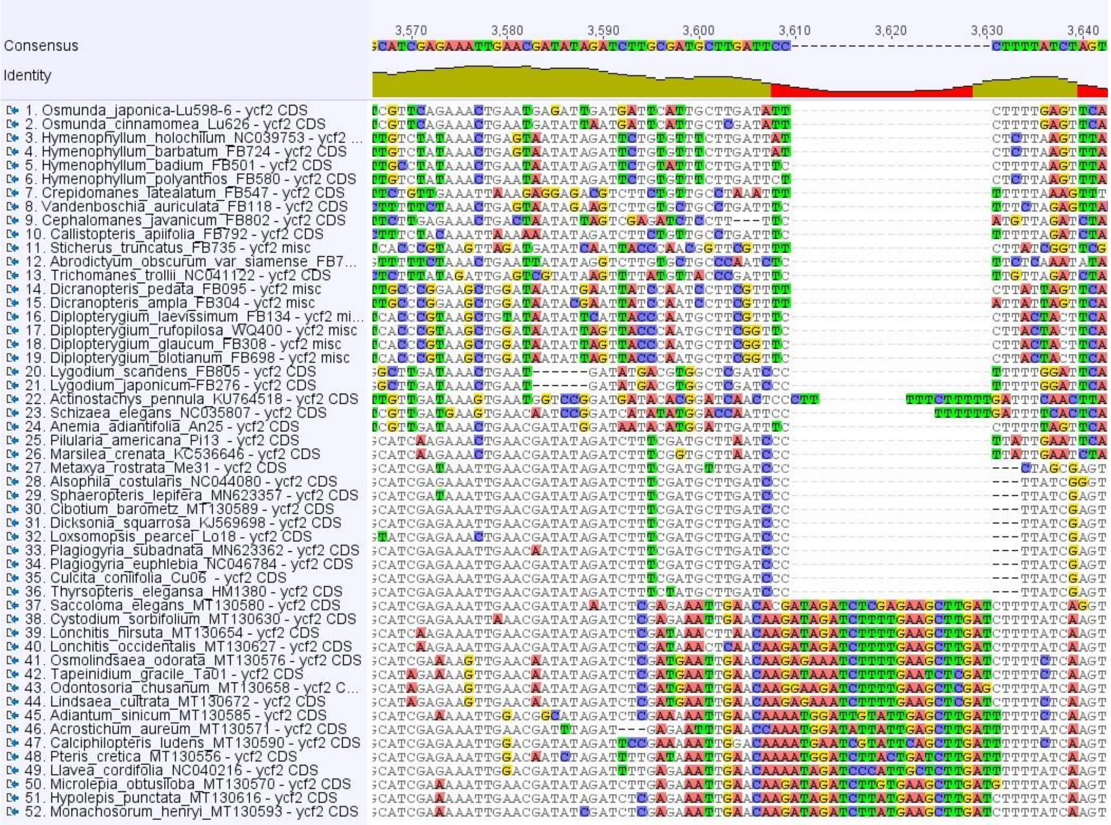

D4: Cyatheales (tree ferns)_ycf2_3610–3633_-3 bp

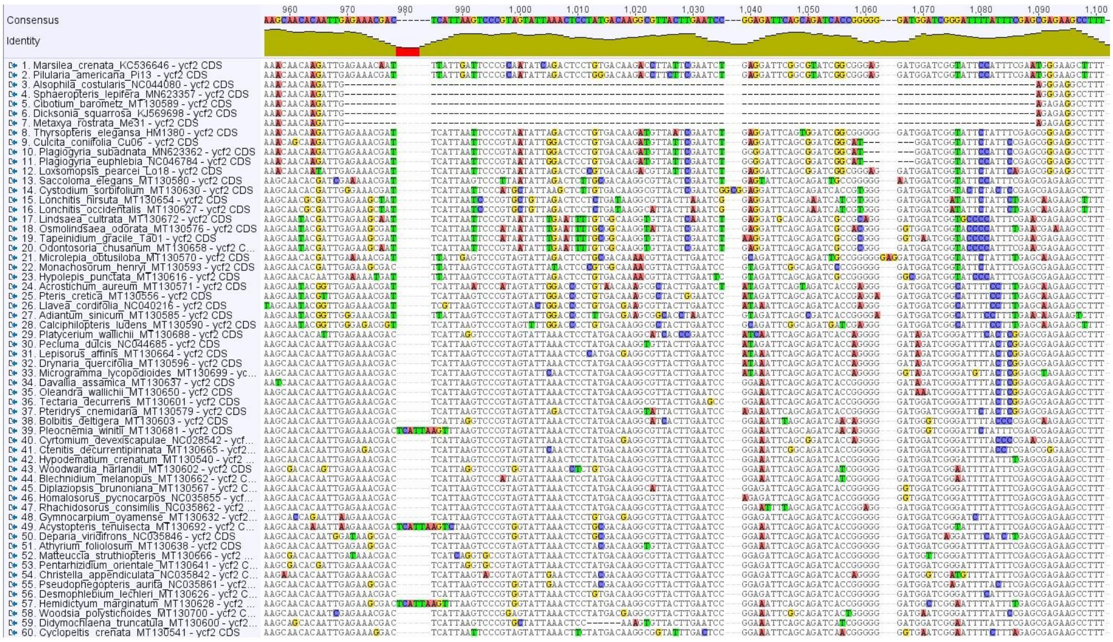

D5: Cibotiaceae, Metaxyaceae, Cyatheaceae, and Dicksoniaceae_ycf2_970–1089_-108 bp

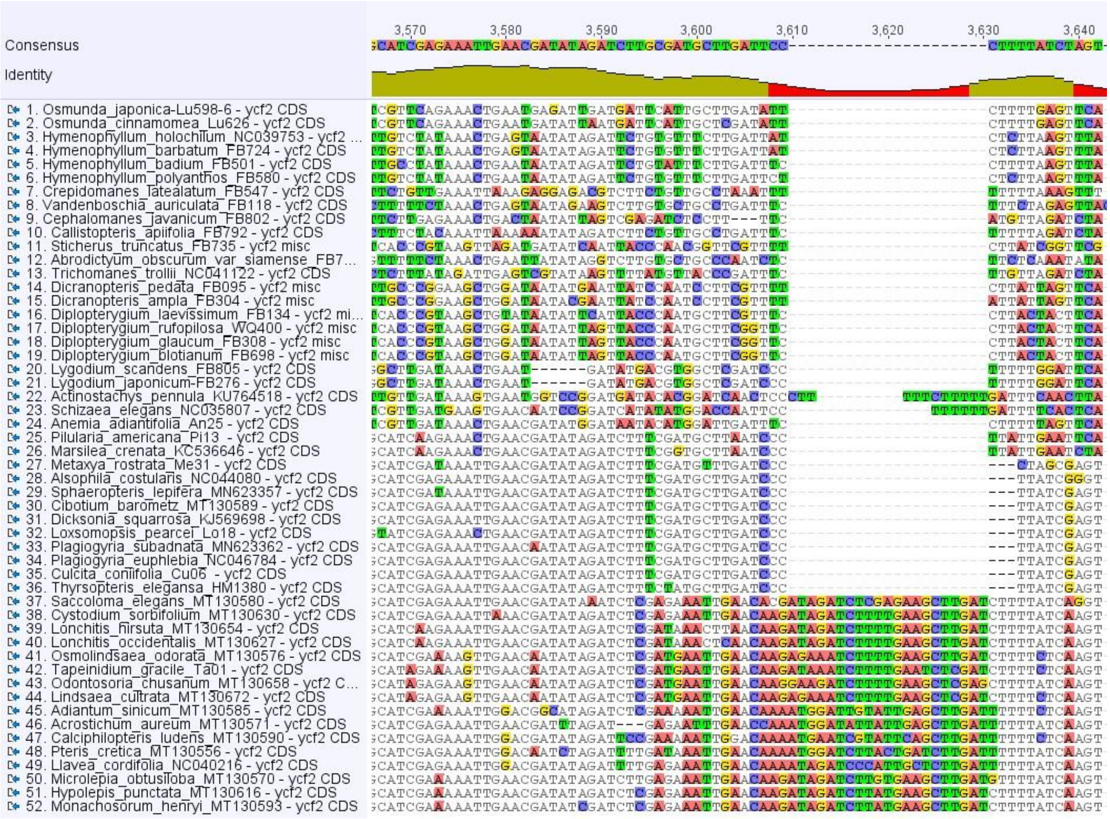

D6: Polypodiales_ycf2_3610–3633_+24 bp

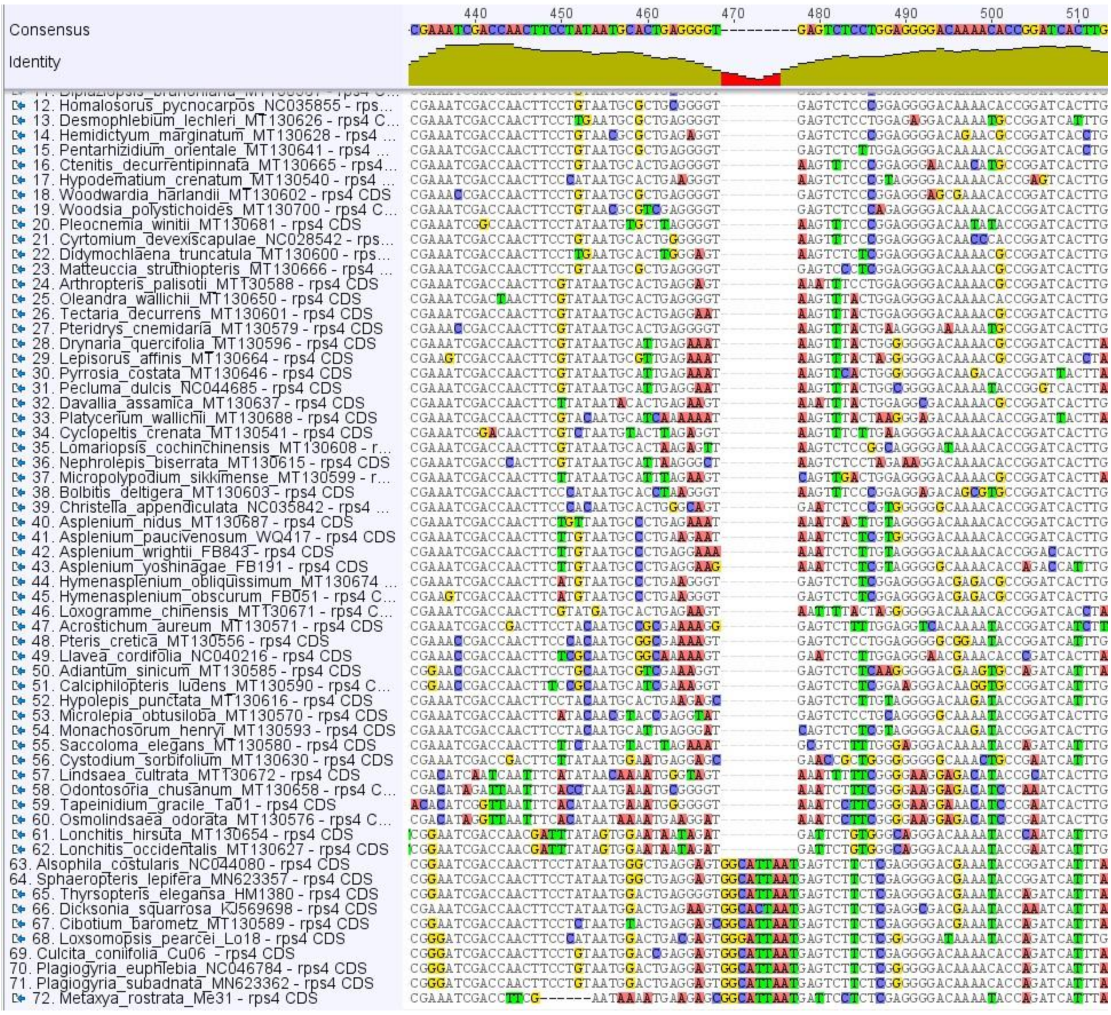

D7: Polypodiales_rps4_469–477_-9 bp

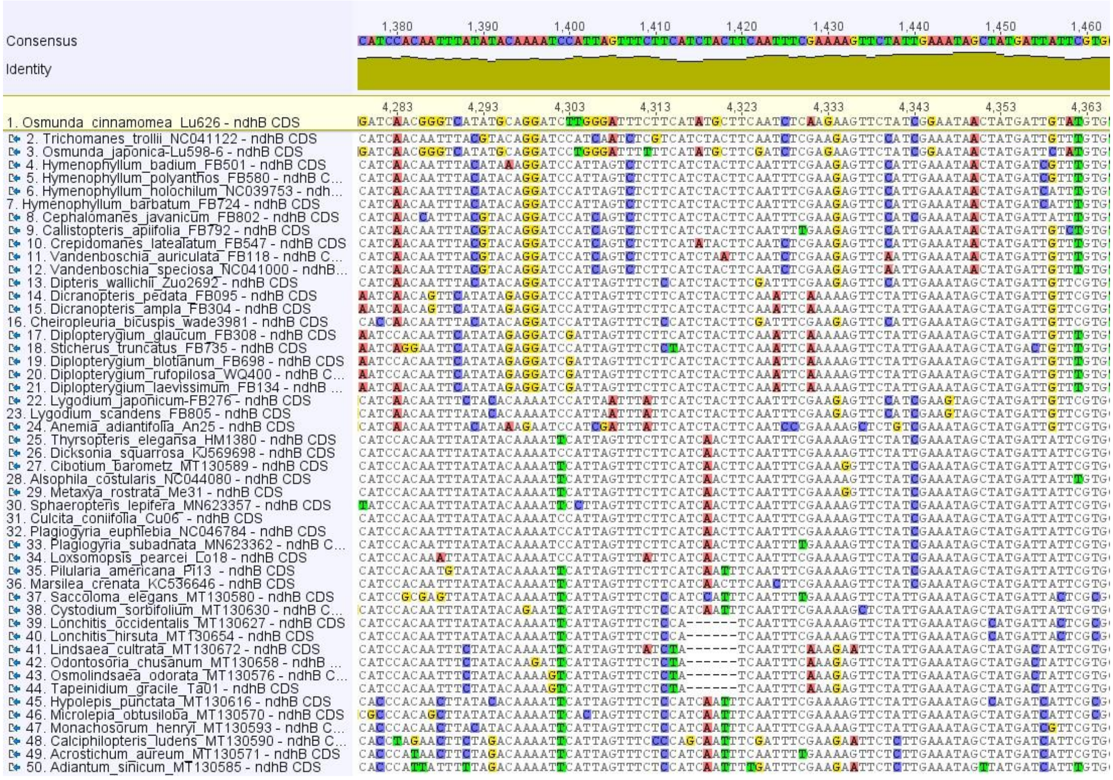

D8: Lindsaeaceae and Lonchitidaceae_ndhB_1414–1419_-6 bp

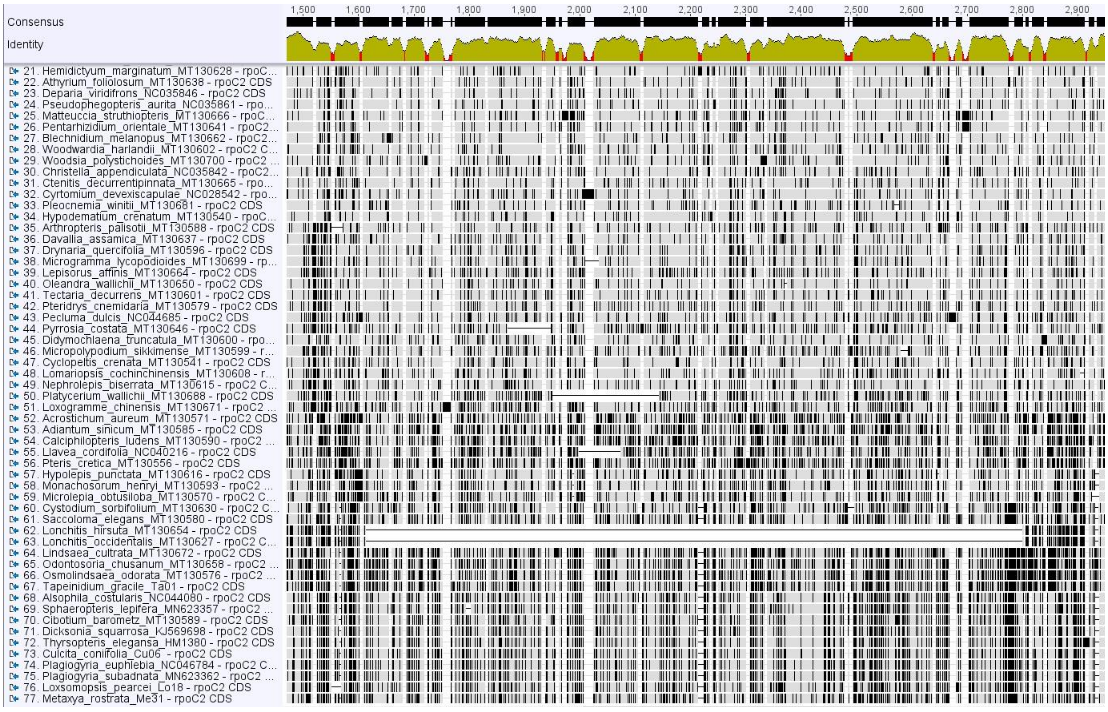

D9: Lonchitidaceae_rpoC2_1615–2799_-1032 bp

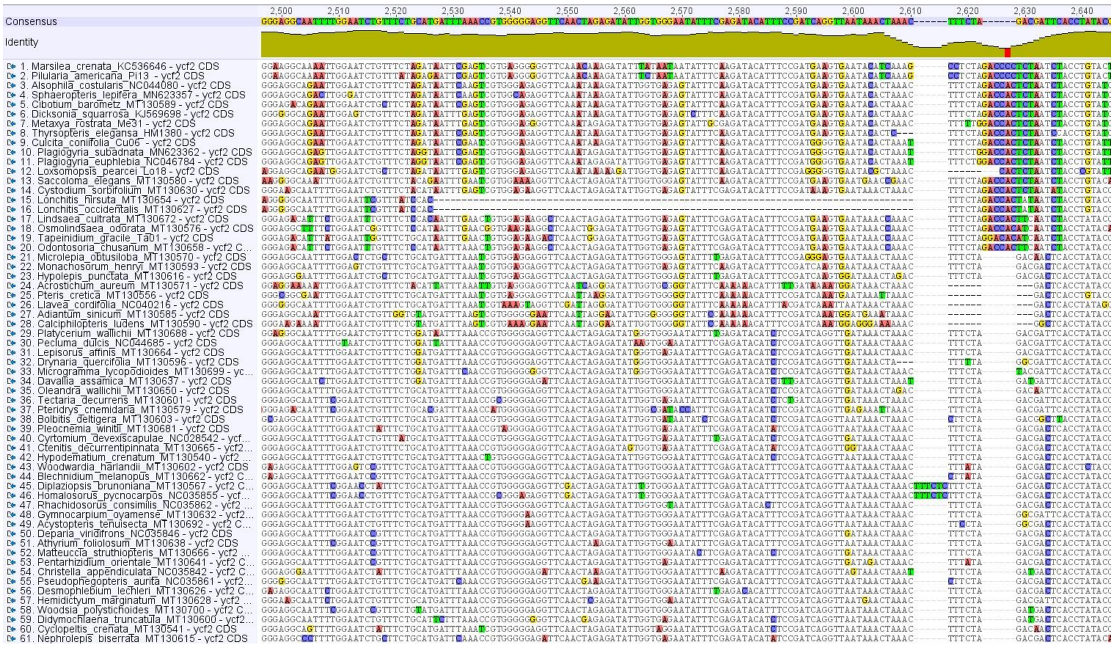

D10: Lonchitidaceae_ycf2_2527–2610_-84 bp

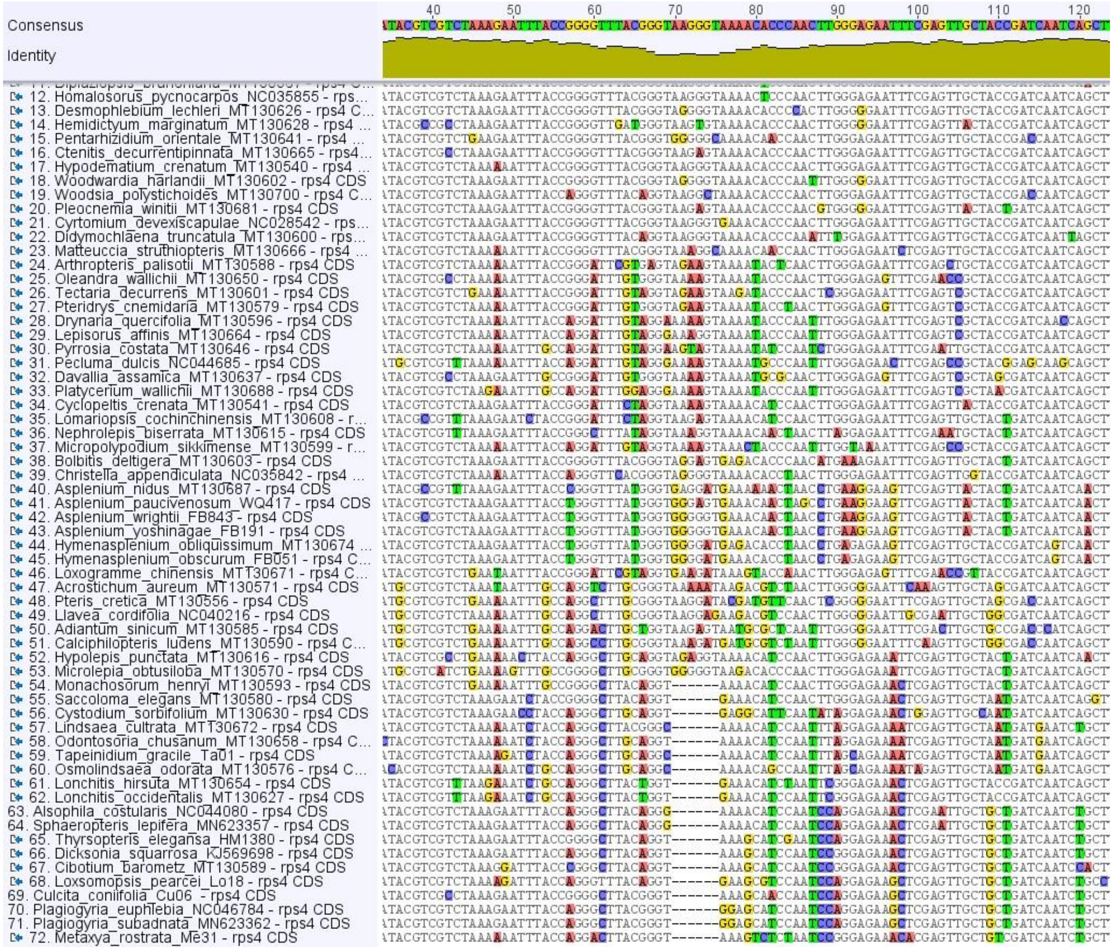

D11: Dennstaedtiaceae, Pteridaceae, and eupolypods_rps4_70–75_+6 bp

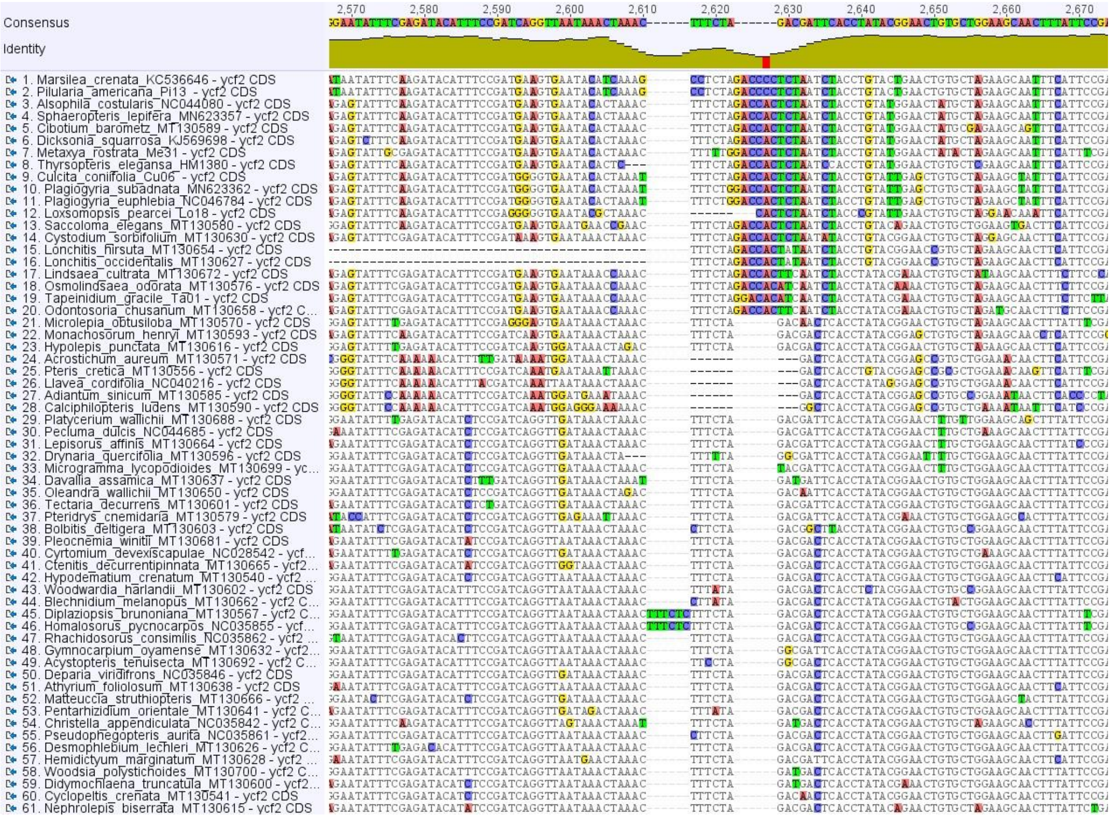

D12: Dennstaedtiaceae, Pteridaceae, and eupolypods_ycf2_2610–2632_-6 bp

D14: Pteridaceae_ycf2_2610–2632_-9 bp

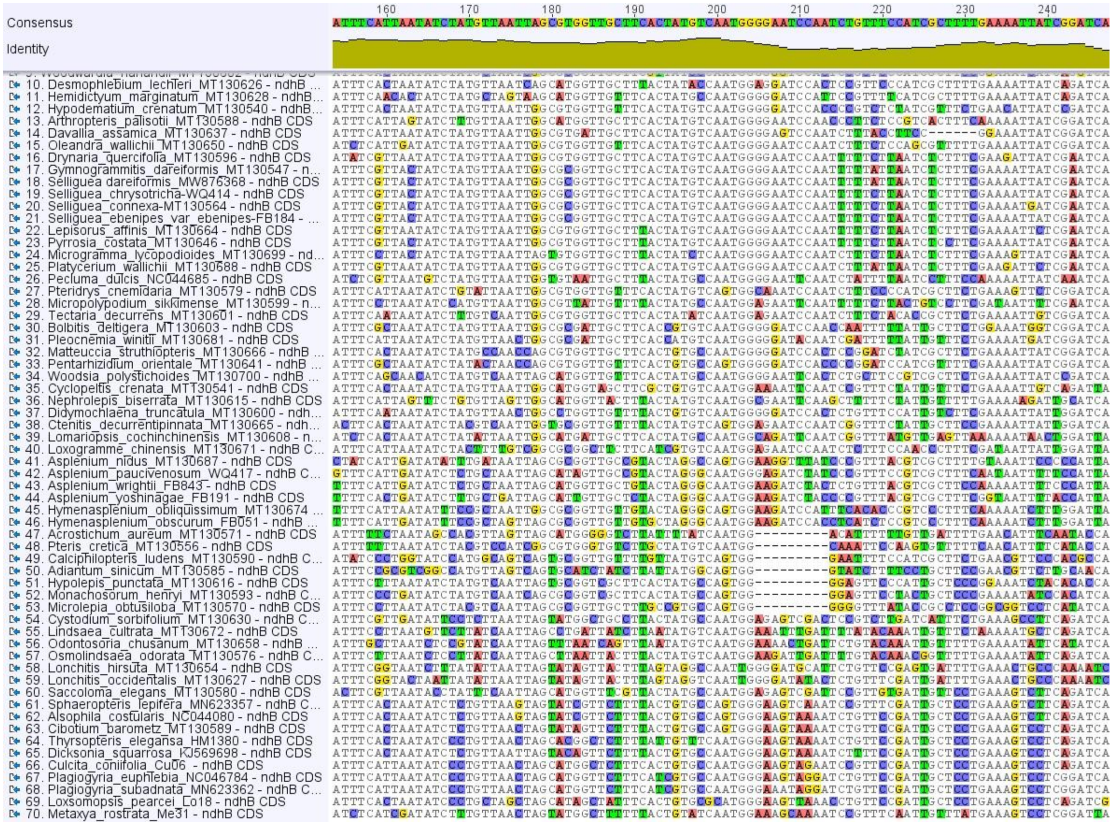

D13: Dennstaedtiaceae and Pteridaceae_ndhB_205–213_-9 bp

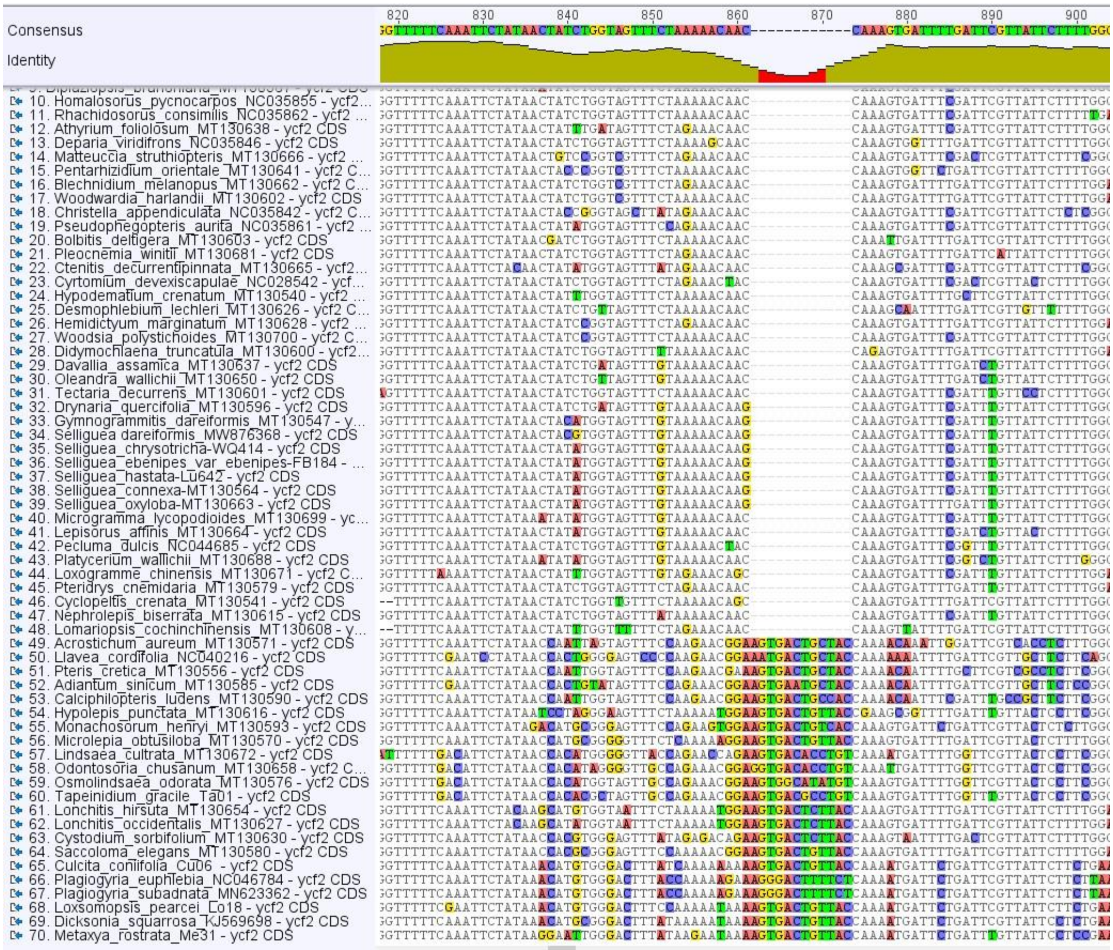

D15: eupolypods_ycf2_862–873_-12 bp

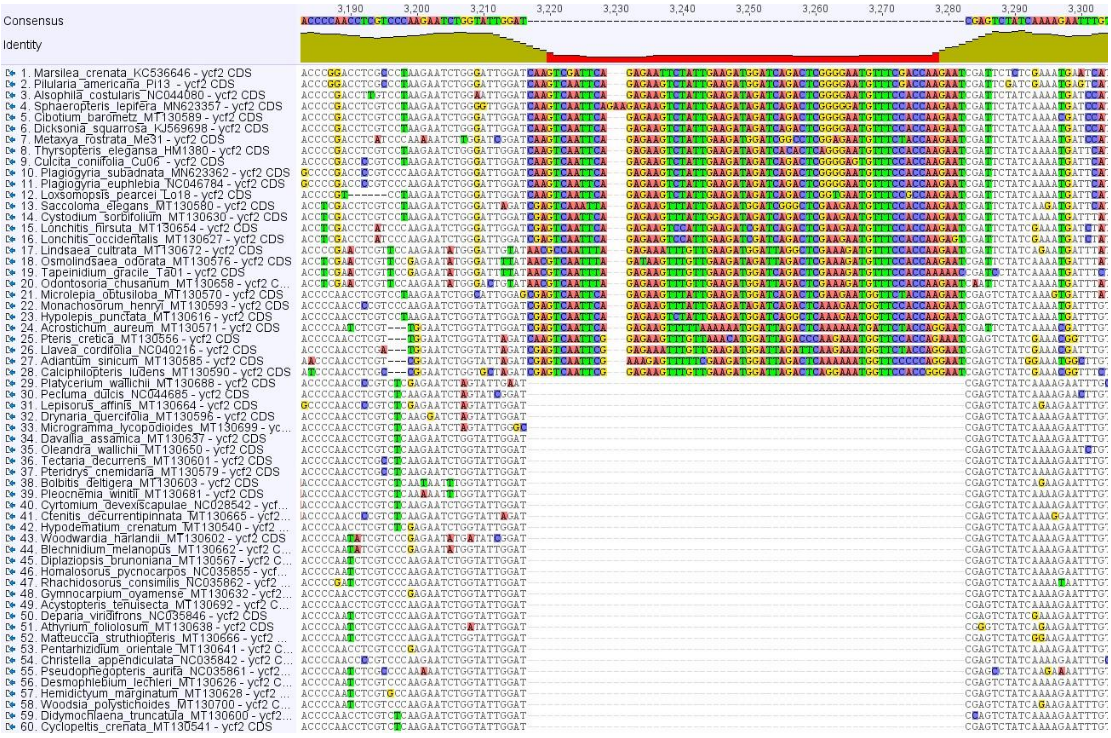

D16: eupolypods_ycf2_3217–3282_-63 bp

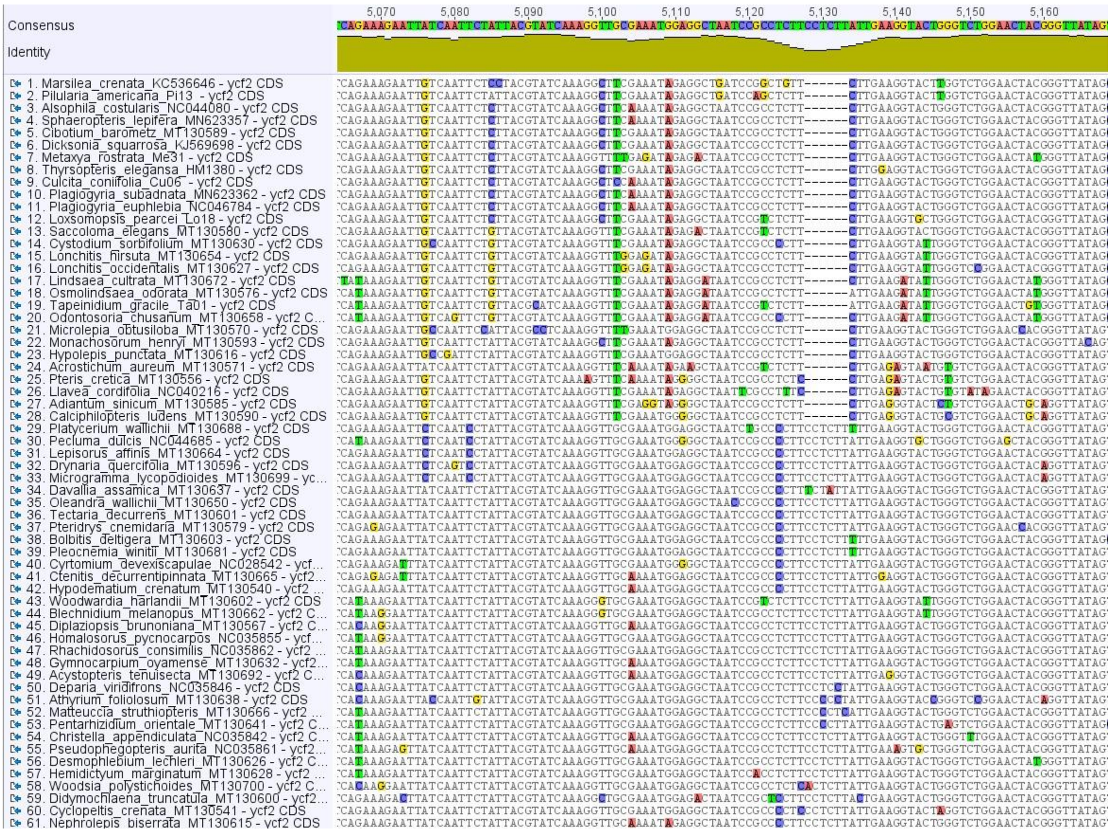

D17: eupolypods_ycf2_5128–5133_+6 bp

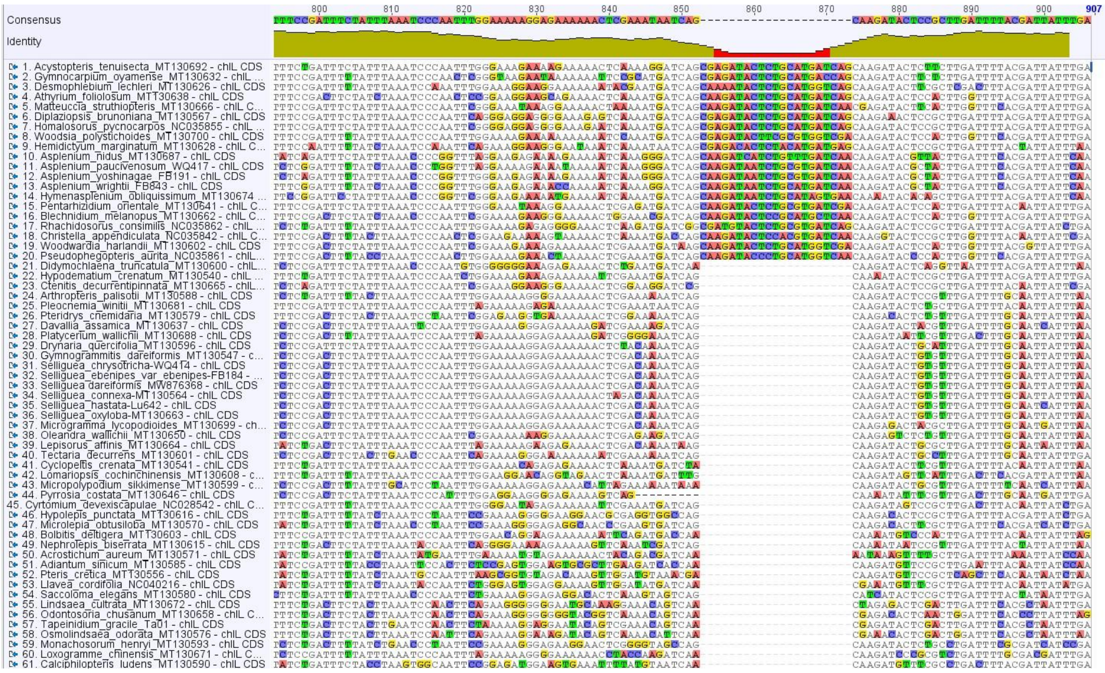

D18: eupolypods II_chlL_853–873_+21 bp

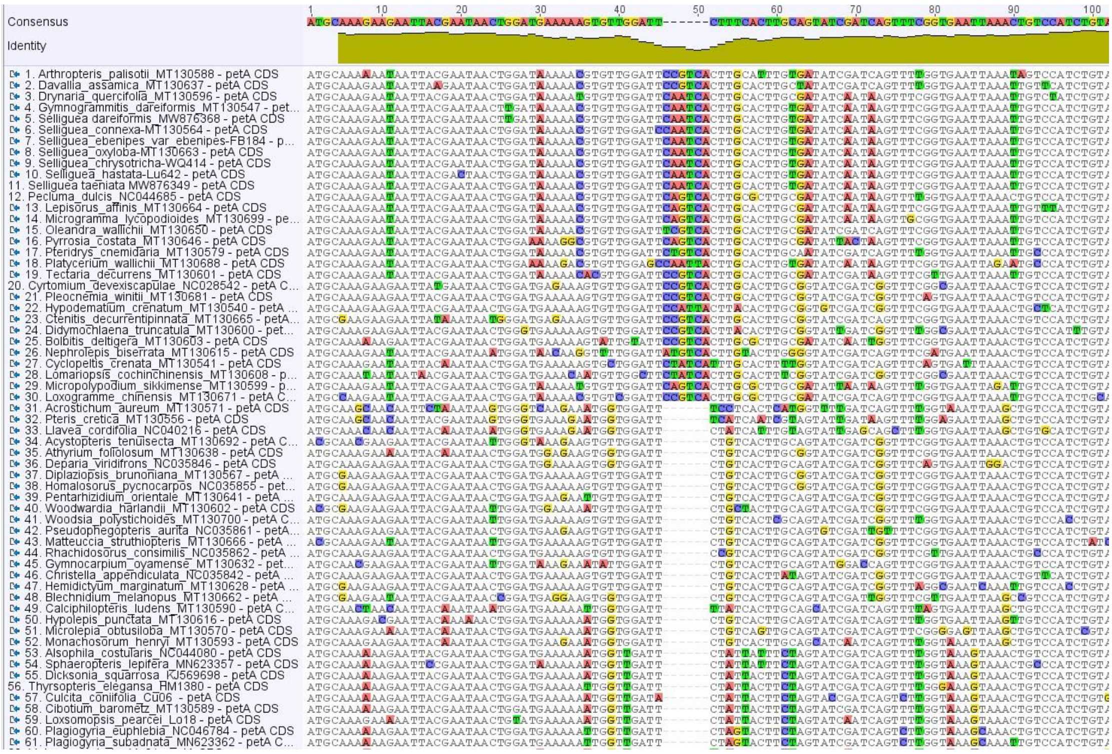

D19: eupolypods I_petA_46–51_+6 bp
