# Supplementary material for: Structural Variation of Plastomes Provides Key Insight Into the Deep Phylogeny of Ferns
Source: Front Plant Sci. 2022 May 2;13:862772. doi: 10.3389/fpls.2022.862772 (PMC9134734; doi:10.3389/fpls.2022.862772)
Supplement: Supplementary file 2 [file Image_1.pdf]

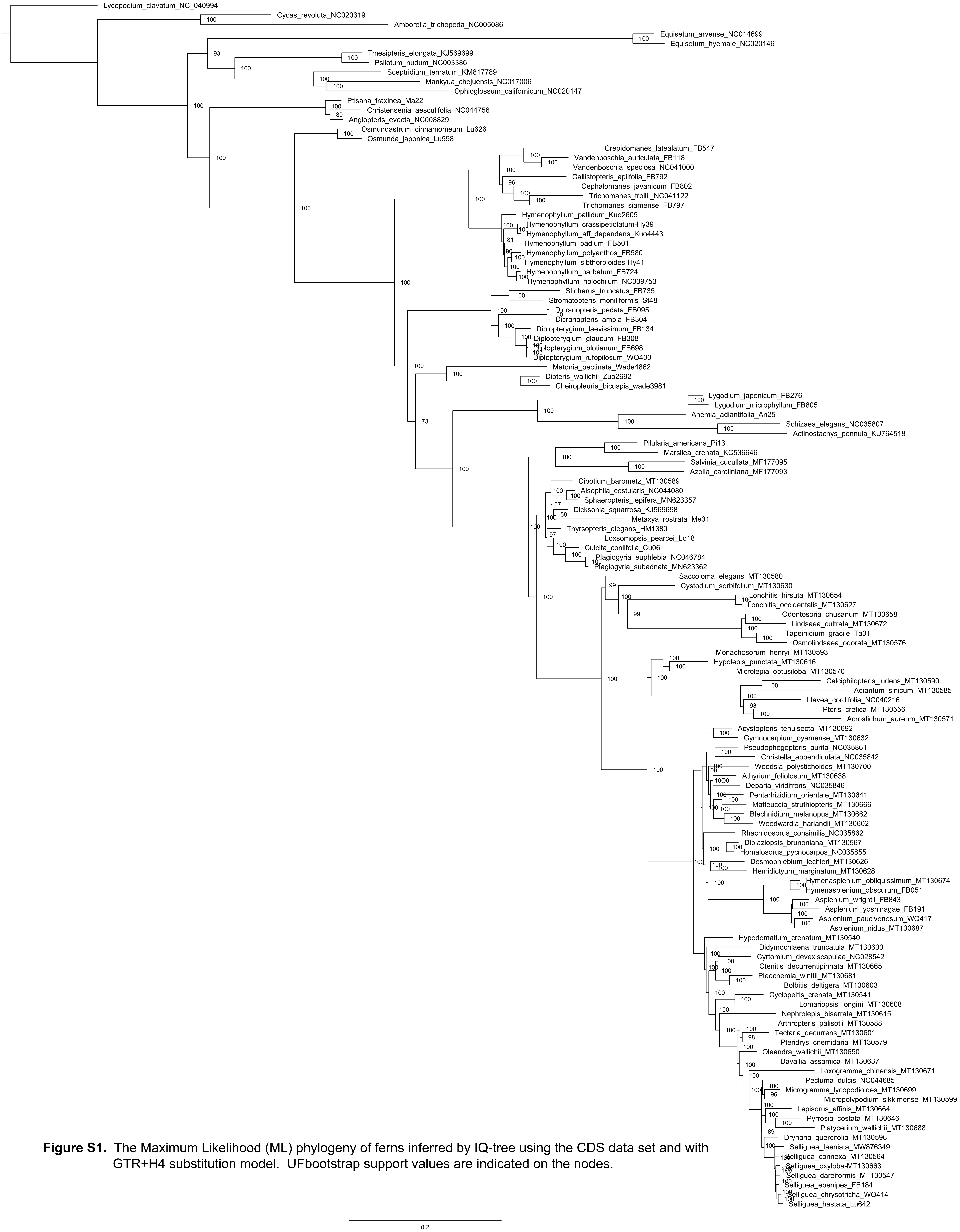

Figure S1. The Maximum Likelihood (ML) phylogeny of ferns inferred by IQ-tree using the CDS data set and with GTR+H4 substitution model. UFbootstrap support values are indicated on the nodes.

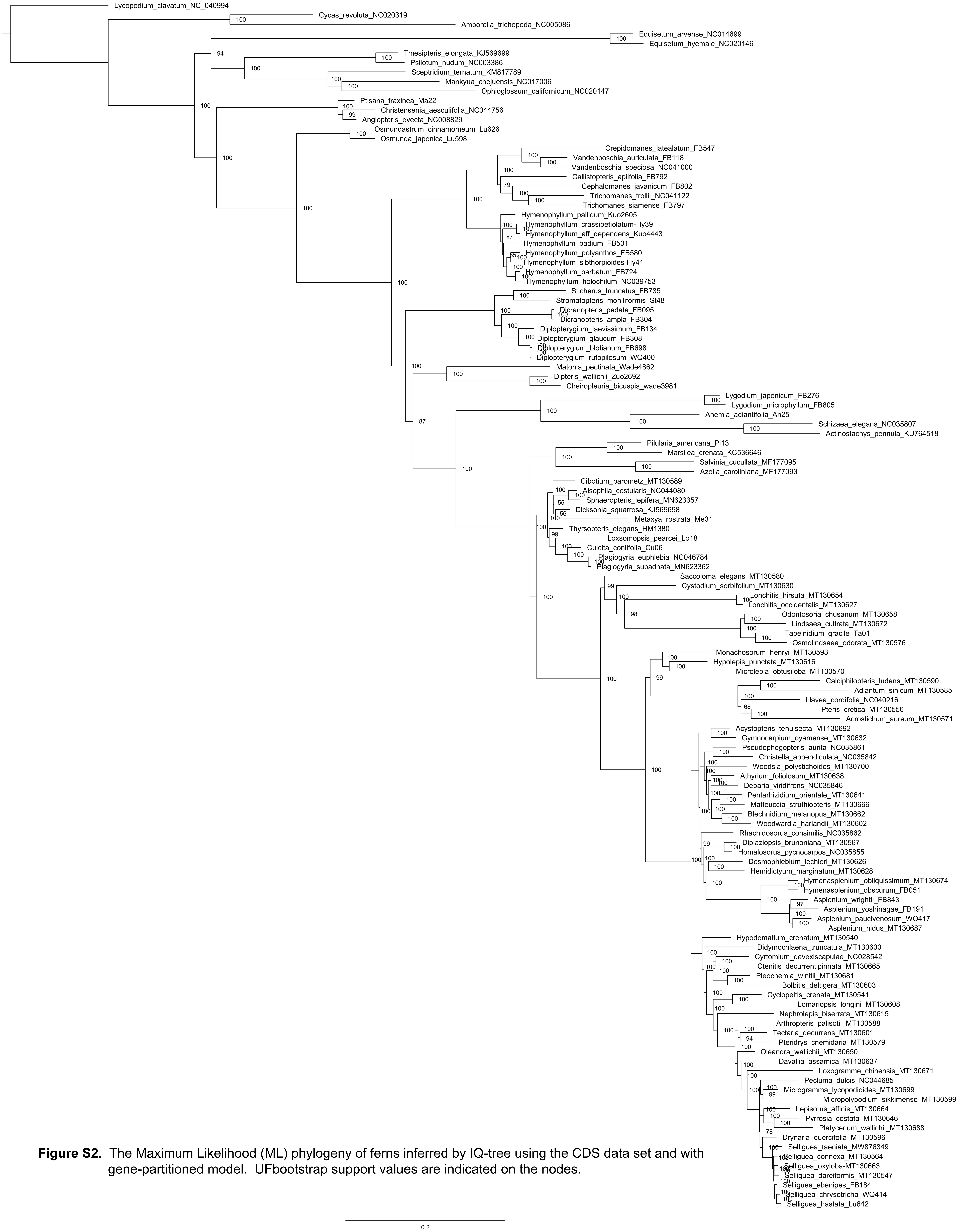

Figure S2. The Maximum Likelihood (ML) phylogeny of ferns inferred by IQ-tree using the CDS data set and with gene-partitioned model. UFbootstrap support values are indicated on the nodes.

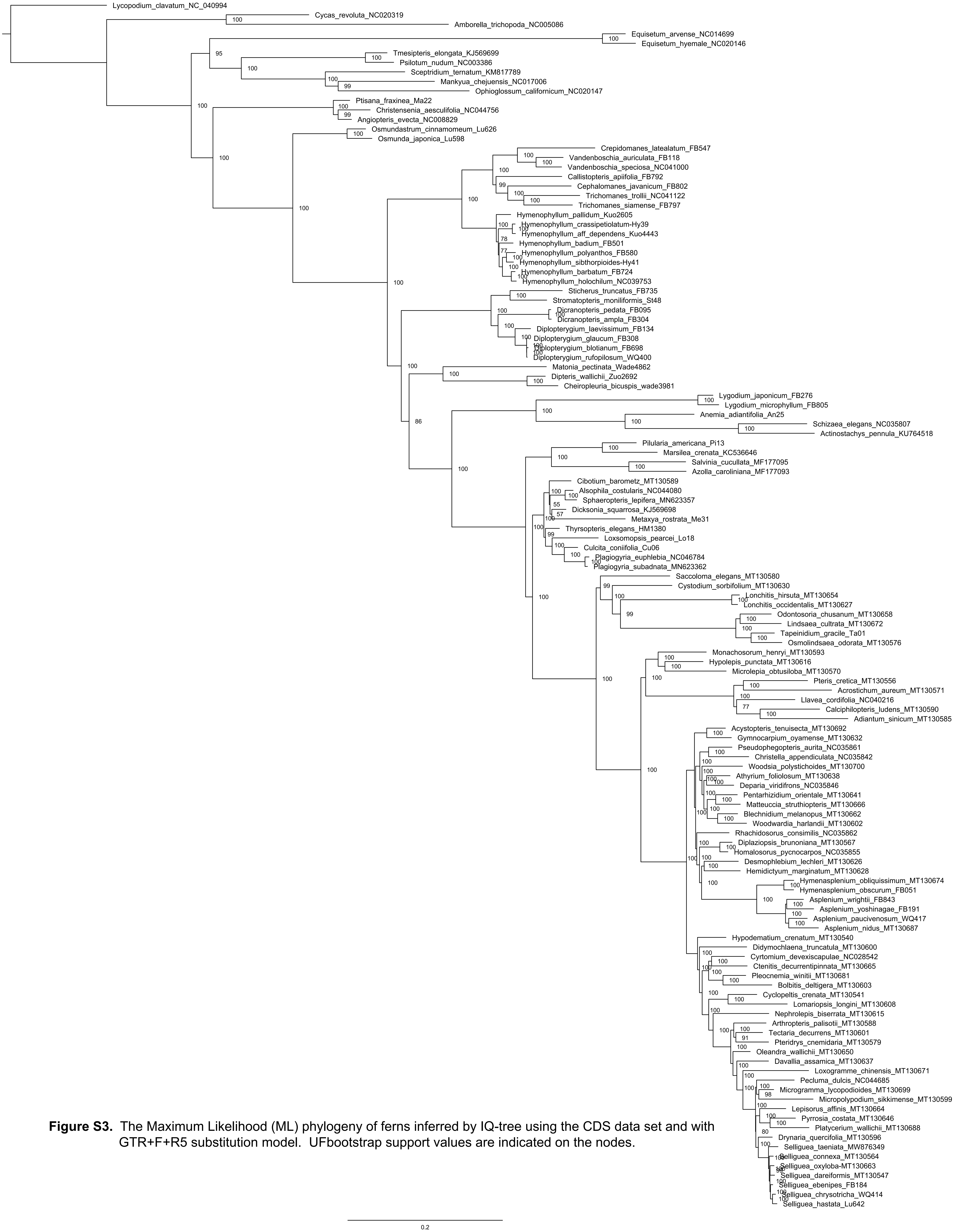

Figure S3. The Maximum Likelihood (ML) phylogeny of ferns inferred by IQ-tree using the CDS data set and with GTR+F+R5 substitution model. UFbootstrap support values are indicated on the nodes.

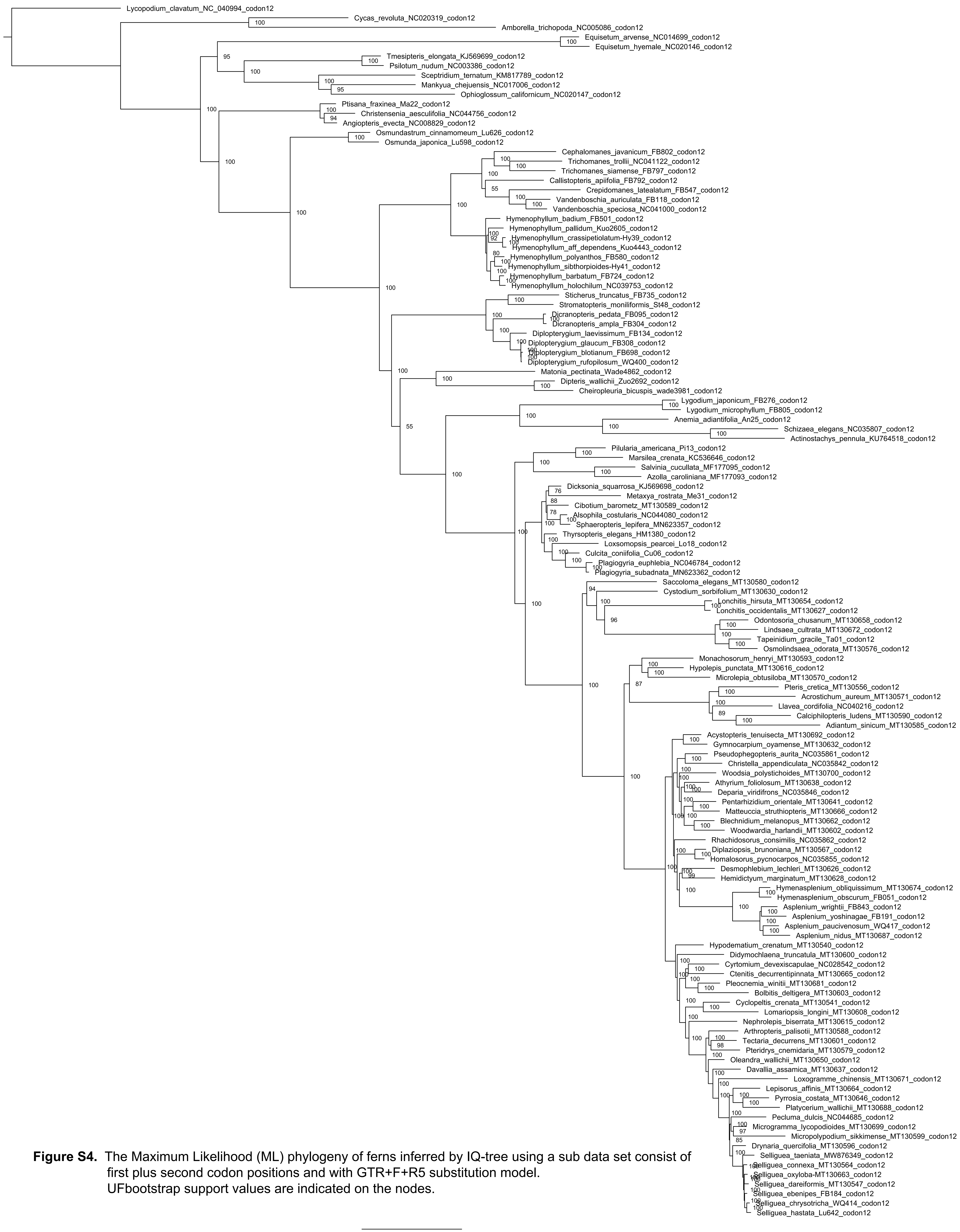

Figure S4. The Maximum Likelihood (ML) phylogeny of ferns inferred by IQ-tree using a sub data set consist of first plus second codon positions and with GTR+F+R5 substitution model. UFbootstrap support values are indicated on the nodes.

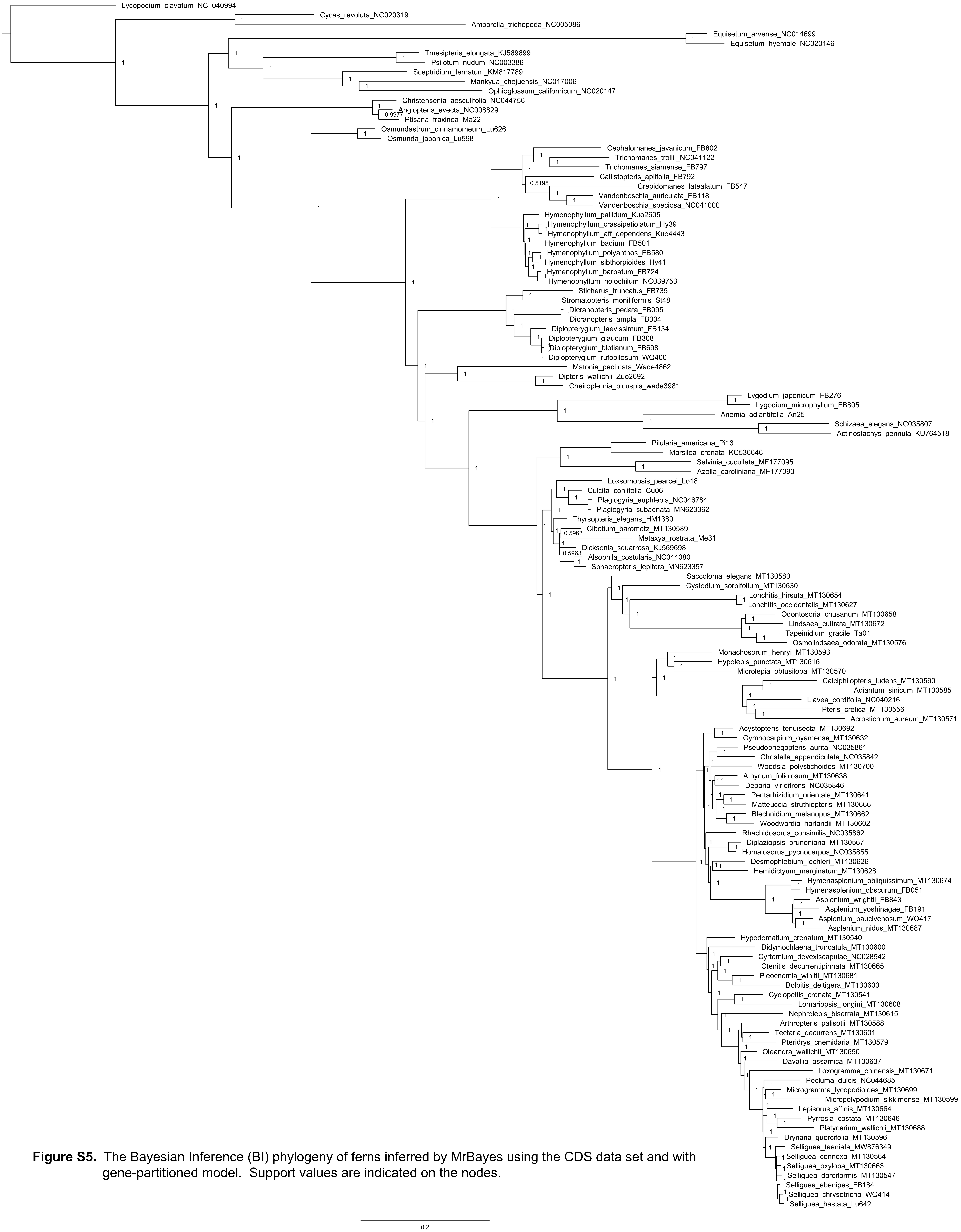

Figure S5. The Bayesian Inference (BI) phylogeny of ferns inferred by MrBayes using the CDS data set and with gene-partitioned model. Support values are indicated on the nodes.
